# Effects of multiple sources of genetic drift on pathogen variation within hosts

**DOI:** 10.1371/journal.pbio.2004444

**Published:** 2018-03-28

**Authors:** David A. Kennedy, Greg Dwyer

**Affiliations:** 1 Center for Infectious Disease Dynamics, Department of Biology, The Pennsylvania State University, University Park, Pennsylvania, United States of America; 2 Department of Ecology and Evolution, University of Chicago, Chicago, Illinois, United States of America; ETH Zurich, Switzerland

## Abstract

Changes in pathogen genetic variation within hosts alter the severity and spread of infectious diseases, with important implications for clinical disease and public health. Genetic drift may play a strong role in shaping pathogen variation, but analyses of drift in pathogens have oversimplified pathogen population dynamics, either by considering dynamics only at a single scale—such as within hosts or between hosts—or by making drastic simplifying assumptions, for example, that host immune systems can be ignored or that transmission bottlenecks are complete. Moreover, previous studies have used genetic data to infer the strength of genetic drift, whereas we test whether the genetic drift imposed by pathogen population processes can be used to explain genetic data. We first constructed and parameterized a mathematical model of gypsy moth baculovirus dynamics that allows genetic drift to act within and between hosts. We then quantified the genome-wide diversity of baculovirus populations within each of 143 field-collected gypsy moth larvae using Illumina sequencing. Finally, we determined whether the genetic drift imposed by host–pathogen population dynamics in our model explains the levels of pathogen diversity in our data. We found that when the model allows drift to act at multiple scales—including within hosts, between hosts, and between years—it can accurately reproduce the data, but when the effects of drift are simplified by neglecting transmission bottlenecks and stochastic variation in virus replication within hosts, the model fails. A de novo mutation model and a purifying selection model similarly fail to explain the data. Our results show that genetic drift can play a strong role in determining pathogen variation and that mathematical models that account for pathogen population growth at multiple scales of biological organization can be used to explain this variation.

## Author summary

The genetic diversity of a pathogen population can strongly influence disease severity and spread. High genetic variation within hosts can lead to severe disease symptoms in individuals and rapid disease transmission in populations. But what factors determine this genetic diversity? Many factors might play roles, including natural selection, mutation, and genetic drift (the randomness caused by chance events in small populations). The relative importance of these factors is unknown because diversity is presumably shaped by dynamics that occur at multiple scales (within and between hosts) and each scale is typically studied independently. We studied the combined impact of dynamics at multiple scales by constructing a mathematical model of pathogen dynamics that simultaneously describes both within-host and between-host disease dynamics. By combining scales, our approach allowed us to ask which factors most strongly influence pathogen genetic diversity. We compared our model to data from natural gypsy moth baculovirus infections. Although pathogen diversity is often assumed to be strongly shaped by natural selection, we found that genetic drift acting at multiple scales provided a better explanation for our data. This suggests that genetic drift plays a key role in shaping pathogen diversity within hosts, which in turn has implications for how we might better combat problematic pathogen evolution, such as the evolution of drug resistance.

## Introduction

Pathogen genetic variation can have important consequences for human health in both clinical and epidemiological settings [1]. In particular, high variation within hosts can lead to severe disease symptoms within individuals and rapid disease transmission within populations [2, 3]. An understanding of the mechanisms determining pathogen variation might therefore lead to novel interventions, reducing the toll of infectious diseases. The development of such an understanding requires a quantification of the effects of population processes on pathogen genetic variation, in turn requiring mathematical models that relate population processes to genetic change.

Such models, however, tend to greatly simplify pathogen biology. Selection–mutation models, for example, often assume that pathogen populations are effectively infinite [4]. Models that allow pathogen population sizes to be finite typically neglect pathogen population processes, either within hosts in acute infections [5] or between hosts in chronic infections [6]. Models that attempt to capture both of these scales of disease dynamics have assumed either that pathogen population growth within hosts is very simple [7] or that pathogen bottlenecks at transmission are complete [7–9], so that every infection begins as a clonal lineage. These simplifications could strongly alter conclusions about the effects of genetic drift on pathogen diversity and indeed have been highlighted as key challenges in phylodynamic inference [10].

Genetic drift is a change in an allele’s frequency due to the chance events that befall individuals. The effects of drift are therefore strongest in small populations, in which a few events can have a large impact [11]. The high population sizes typical of severe infections have led some authors to argue that drift has little effect on pathogens [12, 13], but pathogen population sizes typically fluctuate by several orders of magnitude over the course of infection and transmission. It therefore seems likely that, when pathogen populations are small and variable, pathogen genetic variation will be strongly affected by drift. Indeed, analyses that allow for finite population sizes have shown that drift has at least weak effects on some pathogens [6].

New infections are typically initiated by small pathogen population sizes within hosts [14], leading to bottlenecks at the time of transmission that may drive genetic drift. Pathogen population sizes within hosts can also remain small for long periods following exposure [15]. In small populations, chance events such as the timing of reproduction can strongly influence population growth, a phenomenon known as "demographic stochasticity" [16]. When the effects of demographic stochasticity are strong, chance may allow some virus strains to replicate and survive while others go extinct, providing a second source of genetic drift that we refer to as "replicative drift." Note that we use the term "strain" to mean a population of pathogen particles that have identical genetic sequences.

Many previous studies of genetic drift in pathogens have focused only on population processes that operate within hosts, either during experiments with model organisms or during the treatment of human patients [14, 17]. Pathogen variation in nature, however, is also affected by processes that operate at the host population level, such as fluctuating infection rates during epidemics [18]. Studies of Ebola [19] and tuberculosis [20], for example, have shown that much of the variation present at the population level often cannot be explained by natural selection and must instead be due to neutral processes that presumably include genetic drift.

Genetic drift in pathogens may therefore be driven by population processes at multiple scales. These multiple scales can be incorporated into a single framework by constructing "nested" models, in which submodels of within-host pathogen population growth are nested in models of between-host pathogen transmission [21]. The computing resources necessary to analyze such complex models have only become available recently, however, and so it has not been clear whether sufficient data exist to test nested models of drift [22]. Indeed, even for models that assume that the strength of drift is constant across hosts, robust tests of the model predictions require both genetic data and mechanistic epidemiological models [9], a combination that is rarely available. Whether nested models can be of practical use for understanding pathogen genetic variation in nature is therefore unclear.

For baculovirus diseases of insects, pathogen population processes have been intensively studied at both the host population level [23] and at the individual host level [15]. Baculoviruses cause severe epizootics (epidemics in animals) in many insects [24], including economically important pest species such as the gypsy moth (*Lymantria dispar*) that we study here [25]. Collection and rearing protocols for the gypsy moth have long been standardized [26], and so previous studies of the gypsy moth baculovirus, *Lymantria dispar* multiple nucleopolyhedrovirus (LdMNPV), have produced parameter estimates for both within-host [27] and between-host [28–30] models. Moreover, collection of large numbers of virus-infected individuals is straightforward [25], making it possible to use high-throughput sequencing methods to characterize pathogen diversity across many virus-infected hosts. Here, we use a combination of whole-genome sequencing and parameterized, nested models to quantify the effects of genetic drift on the gypsy moth baculovirus. We show that a mechanistic model of genetic drift can explain variation in this pathogen but only if the model takes into account the effects of drift at multiple scales of biological organization.

## Results

Sequencing the virus populations from each of 143 field-collected insects showed that there is substantial genetic variation in baculovirus populations between hosts. We generated consensus sequences for each of our 143 samples (Section A in S1 Text), and comparisons between consensus sequences identified 712 segregating sites at the between-host scale (defined as sites where alternative variants were the consensus in more than 6 samples [approximately equal to 5%]). These sites correspond to approximately 0.4% of the genome. Analysis of the variation at these 712 sites within each sampled virus population showed that these sites were polymorphic in some hosts but not others, which might occur if some hosts were exposed to multiple strains of virus while others were exposed to only a single strain. We summarize genetic variation within hosts using mean nucleotide diversity [31], the probability that 2 randomly selected alleles at a segregating site are different (Section A in S1 Text). Our conclusions were unchanged when we used alternative metrics of diversity, such as the proportion of polymorphic loci, the effective number of alleles, or the relative nucleotide diversity (Section I in S1 Text).

Calculating nucleotide diversity at the 712 segregating sites using the consensus sequences of our 143 samples showed that between-host nucleotide diversity *π* = 0.404. Within individual field-collected hosts, nucleotide diversity at these same sites ranged from 0.002 to 0.284 (mean = 0.072, SD = 0.077, Section B in S1 Text), while over all sites, nucleotide diversity ranged from 0.001 to 0.003, with a mean of 0.001. In Section B of S1 Text, we show that these values imply that a large fraction of nucleotide diversity within hosts can be explained by just 712 segregating sites, or 0.4% of the genome.

Together, these patterns suggest that substantial pathogen diversity within hosts is likely acquired from the exposure of host insects to multiple virus strains. If diversity had instead been generated by de novo mutation, nucleotide diversity between samples would have been less variable (Section E in S1 Text) and polymorphism would have likely been spread across many sites, including sites that were not polymorphic at the population level. Immune system–mediated diversifying selection is also an unlikely explanation because insects lack clonal immune cell expansion [32], because immune cell expansion does not explain why some hosts have substantially more pathogen diversity than others, and because we found no evidence of diversifying selection in our sequence data (Section H in S1 Text). Negative correlations between host families in susceptibility to different pathogen genotypes constitute yet a third unlikely explanation because such correlations are positive in the gypsy moth [33]. The migration of virus or infected larvae from nearby locations with different virus strains similarly cannot explain the data because population structure in the gypsy moth virus is minimal [34] (Section A in S1 Text).

**Fig 1:**
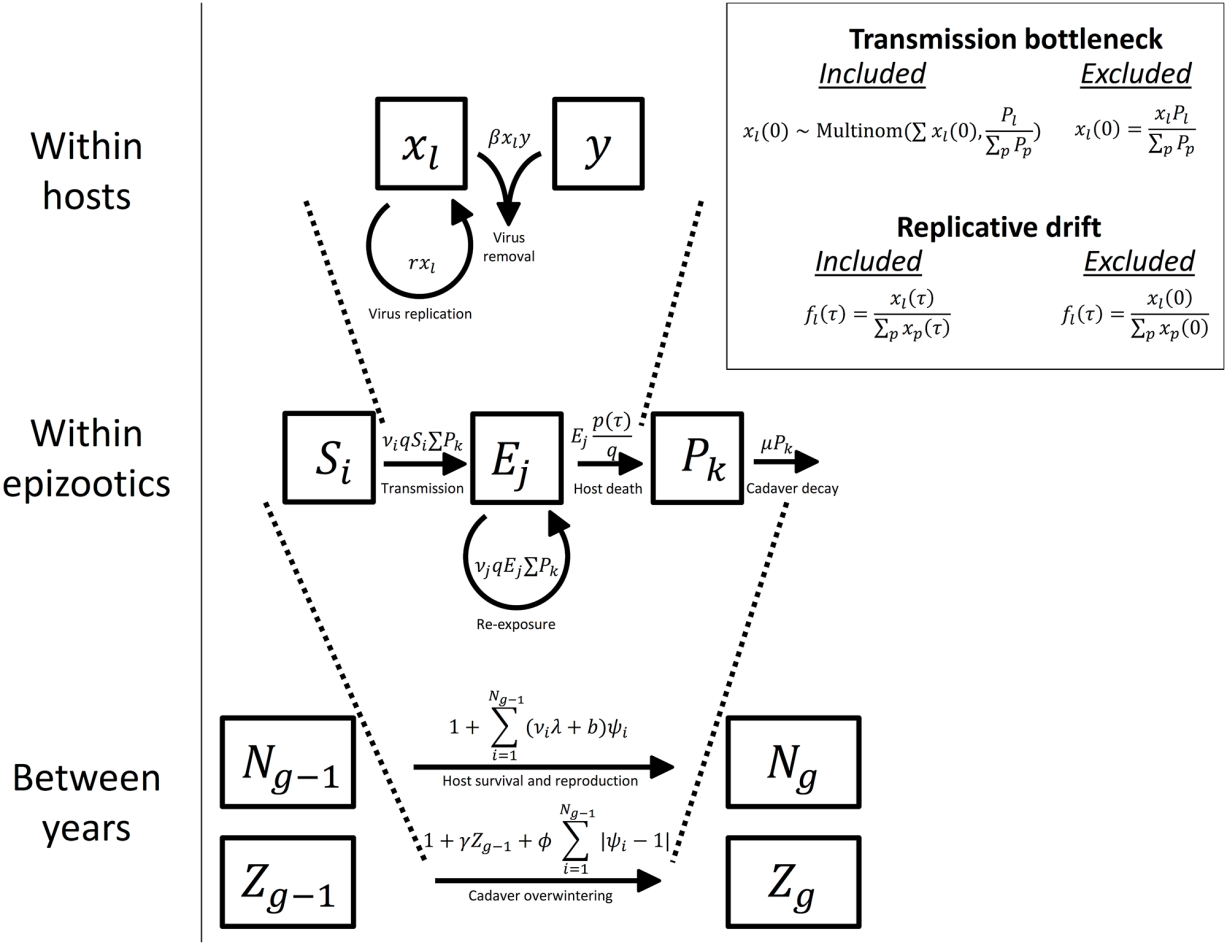
Schematic of the nested model. Bottom: the host population size *N*_*g*_ and the infectious cadaver population size *Z*_*g*_ in generation *g* depend on host and pathogen population sizes in generation *g* − 1 and the disease dynamics in that generation. Following the epizootic, surviving hosts reproduce, and virus-killed cadavers overwinter to start the epizootic in the following year. Middle: the disease dynamics in generation *g* − 1 follow a stochastic SEIR model [35], such that a susceptible host *S*_*i*_ becomes exposed *E*_*j*_ to infectious cadaver *P*_*k*_ at rate *v*_*i*_*q*, where *v*_*i*_ is the risk of exposure for host *i* and *q* is the probability of death given exposure, which arises from the within-host virus dynamics. Note that the "Removed" class *R*, corresponding to inactivated cadavers, is not explicitly shown. The probability of a host dying from virus infection at time *τ* post exposure, *p*(*τ*), is determined by the dynamics of the pathogen within a host. *q* is related to *p*(*τ*) in that $q=\int_{0}^{\infty }p(\tau )d\tau$. Top: within a host, virus particles *x* can reproduce or interact with immune cells *y*, resulting in the removal of both the virus particle and the immune cell. An infection fails to kill the host if all virus particles are cleared so that *x* = 0, but the host dies if the total number of virus particles reaches an upper threshold *C*. Further details are in Section C of S1 Text. To produce a model that lacks replicative drift, we assume that *f*_*l*_(*τ*), the frequency of a virus strain *l* at time of death *τ*, is equal to the frequency of that strain immediately after the time of exposure *f*_*l*_(0). To produce a model that lacks transmission bottlenecks, we assume that the number of copies of a virus strain *l* at the beginning of an infection *x*_*l*_(0) is equal to the total number of virus particles that invade the host ∑_*l*_*x*_*l*_(0) multiplied by the relative frequency of that virus strain in the cadaver that caused exposure *P*_*l*_ / (∑_*p*_*P*_*p*_). In the purifying selection model, host death occurs only if a larva was susceptible to one or more of the virus strains in the cadaver to which it was exposed. If so, the virus strains that the host was susceptible to are released upon host death at frequencies equal to those in the infecting cadaver. SEIR, Susceptible-Exposed-Infectious-Removed.

Genetic drift, however, can explain the data but only if we allow for effects of population processes at multiple scales of biological organization. To explain why, we first use a nested model of pathogen population dynamics (Fig 1) to show how genetic drift in pathogen populations may operate at 3 scales: within hosts, within epizootics, and between years. We then show that the model can only explain the data if it includes effects of drift during both transmission bottlenecks and virus growth within hosts.

**Fig 2:**
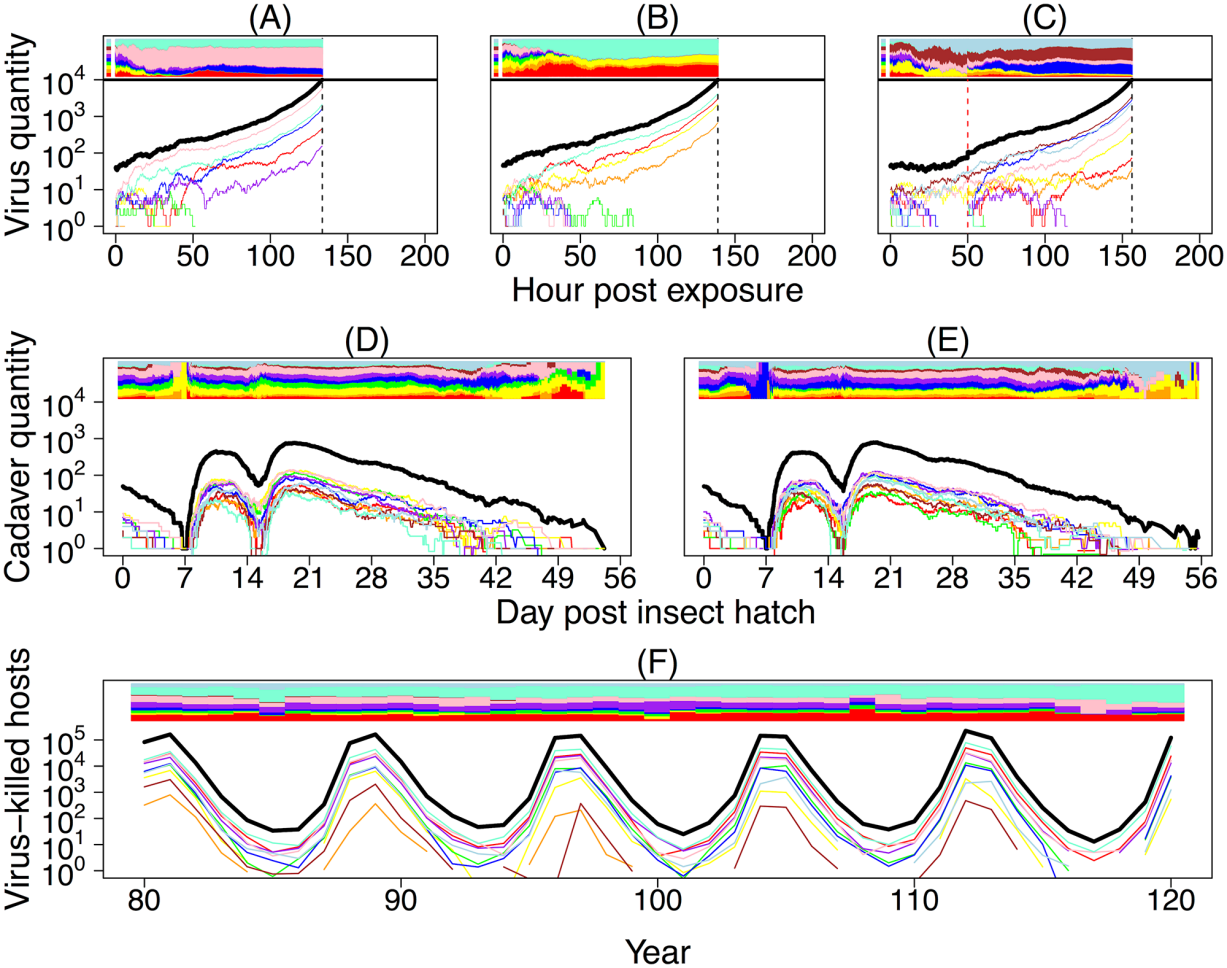
Simulations of the nested model. In all panels A–F, colored curves represent the pathogen population sizes of different virus strains, and the black curve shows the total pathogen population size. The colored bar at the top of each panel shows the relative frequencies of virus strains over time. Panels A–C show 3 realizations of the within-host virus growth model. A reexposure event, marked by a dashed, vertical red line, is also shown in panel C. The top colored panel left of time 0 shows the frequency of virus strains in a cadaver that a host was exposed to at time 0 (and reexposed to at time 50 in panel C). Death occurs when the total number of virus particles within a host hits an upper threshold. To aid visualization, here we set the pathogen population size at host death to be 10^4^, as opposed to the more realistic value of 10^9^ that we use when comparing our models to data. The time of death differs between simulations due to demographic stochasticity in virus growth, and in each simulation it is marked by a dashed, vertical black line. Panels D and E show 2 realizations of our stochastic SEIR-type epizootic model starting from identical initial conditions. Note that the curves here show cadaver quantities rather than virus particles as in panels A–C. Epizootics are initiated by overwintered cadavers that infect emerging larvae. As these cadavers decay, total cadaver quantity drops to low levels, such that the pathogen population is almost entirely composed of virus particles inside living hosts. These hosts then die, initiating future rounds of infections. Panel F shows a realization of our between-generation pathogen model, with trajectories showing the total number of virus-killed hosts in each generation. The frequency of pathogen strains can drift over time, an effect that is particularly noticeable during troughs of infection. SEIR, Susceptible-Exposed-Infectious-Removed.

Simulations of our within-host model show that the combination of transmission bottlenecks and replicative drift can substantially reduce pathogen diversity within hosts (Fig 2A–2C). Demographic stochasticity, which is manifest in the figure as jaggedness in the model trajectories, is strongest shortly after exposure, when the pathogen population size is small. This stochasticity generates variability in the time to host death, and it also drives replicative drift. Comparing this model to a linear birth–death model (Section C in S1 Text) shows that the immune system substantially slows the growth of the virus population early in the infection, which strengthens the effects of replicative drift.

Overwintered virus infects hatchlings during the initial emergence of hosts from eggs, an effect that is apparent in our simulations of the epizootic model (Fig 2D and 2E). After the overwintered virus decays, there is a short period when cadavers are rare, such that the vast majority of virus is present only within exposed larvae. When these exposed larvae die, the virus that they release is transmitted to new larvae feeding on foliage. During this time, the relative frequencies of different virus strains consumed by larvae can fluctuate strongly due to the drift that occurs when cadavers are rare. Low densities of cadavers can thus alter the relative frequency of strains within hosts. In the figure, the initial host population consists of more than 10,000 hosts, reflecting the high densities at which baculovirus epizootics occur in insect populations in nature [24]. Demographic stochasticity nevertheless influences the composition of virus strains near the end of the epizootic, when the pathogen population begins to die out, in turn allowing drift to influence which virus strains cause infections within hosts.

Over longer time periods, fluctuations at the population scale (Fig 2F) produce host–pathogen cycles that match the dynamics of gypsy moth outbreaks in nature [29, 36]. These large fluctuations can drive changes in the relative frequency of pathogen strains, especially when pathogen population sizes and overall infection rates are at their lowest, in the troughs between host population peaks. Host–pathogen population cycles in our model thus further strengthen the effects of genetic drift on the pathogen.

Our combined model shows that drift can act both within and between hosts and at timescales ranging from hours to decades. One way to test the model would therefore be to collect data on pathogen genotypes over time, to see whether observed changes in genotype frequencies match those predicted by the model. However, logistical constraints common across many infectious disease systems, such as the long time intervals between epizootics and the difficulty of collecting samples when infection is rare, precluded the collection of such extensive data. We therefore instead used our model to predict the expected distribution of nucleotide diversity across hosts during an epizootic, and we compared this distribution to the corresponding distribution from our sequence data.

To test whether the data can be explained equally well by models that neglect one or more sources of drift, we also tested models that eliminated replicative drift or that eliminated both replicative drift and transmission bottlenecks. Note that it is not possible to construct a model that includes replicative drift but not transmission bottlenecks because replicative drift requires that virus population sizes be integer values, and forcing the virus population to be an integer value necessarily imposes a form of bottleneck. Also, to test whether the data are better explained by selection than by drift, we constructed a model that allows for purifying selection to act within hosts but that lacks both replicative drift and transmission bottlenecks.

**Fig 3:**
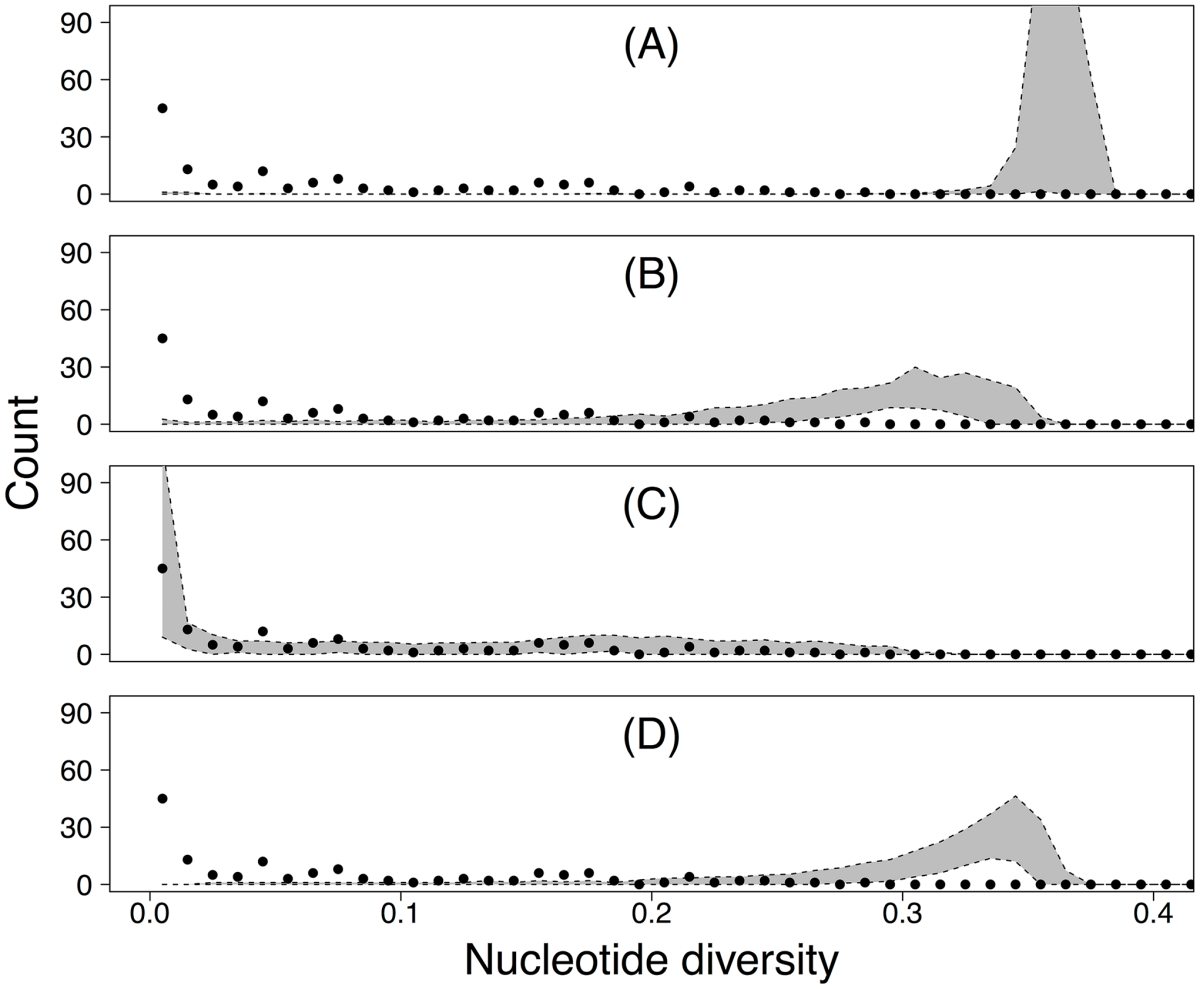
Fit of models to data. Comparison of the predictions of our models (gray-shaded areas, showing 95% CIs of model realizations) to the distribution of nucleotide diversity within 143 individual infected hosts calculated from our sequence data (black dots show data on nucleotide diversity within hosts). Panel A shows the predictions of a model that lacks both transmission bottlenecks and replicative drift, panel B shows the predictions of a model that includes transmission bottlenecks but not replicative drift, panel C shows the predictions of a model that includes both transmission bottlenecks and replicative drift, and panel D shows the predictions of a model that includes purifying selection within hosts but not transmission bottlenecks or replicative drift. Values underlying data points are provided in S1 Data.

These comparisons show that only the model that includes both replicative drift and bottlenecks can explain the data (Fig 3). The model that includes only drift at the host population scale predicts within-host diversity levels that are much higher and much less variable than in the data. The model that includes population scale drift and bottlenecks but not replicative drift as well as the model that includes purifying selection but not transmission bottlenecks or replicative drift both correctly predict that there will be substantial variation across hosts, but they predict diversity levels that are much higher than in the data. In contrast, the model that includes replicative drift and transmission bottlenecks accurately predicts the entire distribution of diversity levels seen in the data. This visual impression is confirmed by differences in the Monte Carlo estimates of the likelihood scores across models (Section F in S1 Text: neutral model with neither bottlenecks nor replicative drift, median log mean likelihood = −503.2; purifying selection model, median log mean likelihood = −353.1; neutral model with bottlenecks but not replicative drift, median log mean likelihood = −266.7; neutral model with both bottlenecks and replicative drift, median log mean likelihood = −63.9). Because no parameters were fit to the diversity data, we do not need a model complexity penalty, but the difference in the number of parameters across models was, in any case, dwarfed by the differences in the likelihood scores. In Section I of S1 Text, we show that the differences in model likelihoods hold when using alternative diversity metrics, and in Sections D and G of S1 Text, we show that our qualitative conclusions are robust across parameter values that determine bottleneck severity and selection intensity.

Our results thus show that a model that accounts for the effects of population processes at multiple scales can explain differences in pathogen variation across hosts in the gypsy moth baculovirus. In contrast, models that simplify the effects of genetic drift by ignoring effects of transmission bottlenecks and replicative drift, or that allow for selection but not within-host drift, cannot explain the diversity of this pathogen. More broadly, because the model parameters were estimated entirely from experimental data on baculovirus infection rates (Section C in S1 Text), we are effectively carrying out cross-validation of the model.

**Fig 4:**
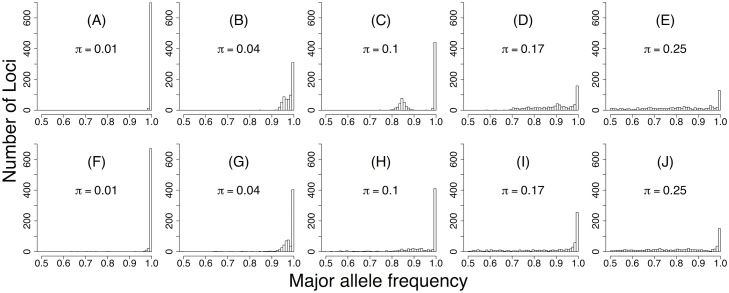
Allele frequencies within hosts. Representative distributions of allele frequencies from individual hosts in our best model (A–E) and in our data (F–J). Each plot shows the distribution of allele frequencies within a single individual at 712 segregating sites, showing only the frequency of the most common allelic variant at each locus within that host. The number on each plot is the mean nucleotide diversity within that particular host. Model plots are aligned with similar data plots. The lack of diversity in panels A and F suggests that the virus population within these hosts consists of only a single virus strain. The bimodal distributions in panels B, C, and G suggest that these virus populations contain exactly 2 virus strains. The high diversity but lack of bimodality in panels D, E, H, I, and J suggests that these virus populations consist of more than 2 virus strains. Values underlying panels F–J are provided in S2 Data.

The highly skewed distribution of nucleotide diversity apparent in our data can therefore be explained by a model that allows for drift at multiple scales and that includes multiple sources of drift within hosts but not by simpler models. In addition, Fig 4 shows that the best model can reproduce entire distributions of diversity within individual hosts. Allele frequency distributions in the model nevertheless tend to have slightly shorter tails and narrower peaks compared to the data. These mild discrepancies may be partially explained by mutations that occurred during viral passaging or during library preparation, but they can also be explained by small biases introduced during the mapping of our short sequence reads to the reference genome (Section J in S1 Text). The data therefore do not reject the model.

**Fig 5:**
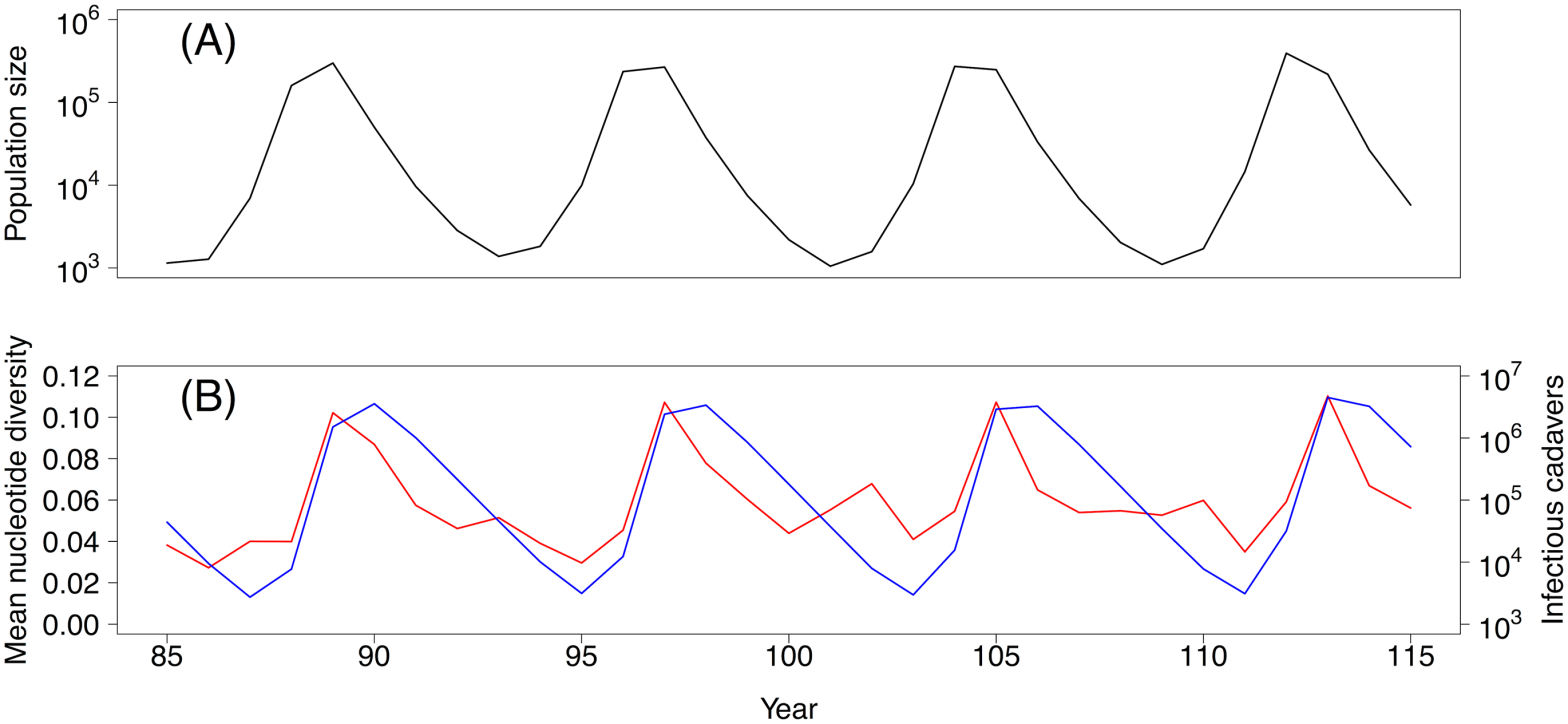
Dynamics over time. Model predictions of the effects of changes in the populations of susceptible and infected hosts on within-host pathogen diversity, over the host–pathogen population cycle. (A) The population size of uninfected hosts. (B) The population size of infectious cadavers (blue) and the mean nucleotide diversity (red).

Our virus samples were collected at times of peak or near-peak gypsy moth densities, which are the only times at which large numbers of larvae can be collected easily, and so the data do not directly show how changes in pathogen population size at the host population scale affect pathogen variation. We therefore used our best model to explore how pathogen variation within hosts will change over the course of the gypsy moth outbreak cycle. Within-host diversity is predicted to be highest just as the host population begins to crash due to the pathogen, after which diversity is predicted to gradually decline until the next outbreak (Fig 5). Reductions in within-host variation due to transmission bottlenecks and replicative drift are thus counterbalanced by increases in within-host variation at the time of host population peaks, due to the high frequency of multiple exposures when host populations are large. Long-term, population scale processes can therefore also strongly affect within-host variation.

## Discussion

A basic prediction of population genetics theory [11] and a fundamental assumption of phylodynamic modeling [18] is that the effects of genetic drift are determined by population processes. Explicit tests of this assumption for infectious diseases, however, are rare. We used a model that was developed and parameterized using nongenetic datasets to show that patterns of genetic diversity in an insect pathogen can be explained by a model that accounts for population processes at multiple scales but not by models that simplify or neglect the effects of drift. Previous work has attempted to infer disease demography and pathogen evolution from genetic data [18]. Here, we instead began with an existing population process model that has already been fit to epidemiological data, and we used it to predict pathogen genetic data. We therefore tested the extent to which disease demography can be used to predict neutral pathogen evolution. By using this approach, we have shown that evolutionary hypotheses can be tested by integrating genetic data and ecological models.

A simple model of purifying selection was not able to explain the patterns of diversity in our data. Models that instead invoke diversifying selection or more complex patterns of host-specific immune selection might provide reasonable explanations for our data, but such models require extra parameters to account for the costs and benefits of alternative alleles, increasing the complexity of the models [37]. Moreover, drift is an inherent property of small populations, and so models that invoke selection should still allow for effects of drift if population sizes are small. In our case, complex models of selection were not needed to explain patterns of diversity, suggesting that the effects of selection on our data are weak relative to the effects of drift. Selection may nevertheless be necessary to explain variation in other pathogens or other datasets. Given that polymorphism has been widely observed in insect baculoviruses [38, 39], our results suggest that baculoviruses present opportunities to understand the relationship between host–pathogen ecology and pathogen diversity.

We have shown that both transmission bottlenecks and replicative drift have detectable impacts on pathogen diversity. Due to the difficulty of separating these effects, previous studies of genetic drift have assumed that bottlenecks are complete [6–8], have ignored impacts of key biological processes such as the host immune response [40], or have summarized the effects of multiple sources of drift with a single parameter—the effective population size *N*_*e*_ [41]. Similarly, estimates of bottleneck size often combine the effects of transmission bottlenecks and replicative drift into a single estimate of the effective bottleneck, biasing estimates of transmission bottleneck size (section D in S1 Text) [40]. Distinguishing between transmission bottlenecks and replicative drift, however, may provide novel insights into disease control strategies. For example, the emergence of resistance to antibiotic drugs might be slowed if drug therapy windows are restricted to periods when the effects of replicative drift are strongest, such as when pathogen populations are small or are turning over rapidly.

To show that both transmission bottlenecks and replicative drift play an important role in shaping pathogen diversity within hosts, we have focused our analysis on common variants that cannot be easily explained by de novo mutation. Additional variation is nevertheless present (Section B in S1 Text). In our case, this other variation occurs at such low levels that it cannot be readily distinguished from sequencing error, but it is almost certainly true that mutation and selection also play roles in shaping total pathogen diversity within hosts. Our argument is therefore not that mutation and selection are unimportant but instead that transmission bottlenecks and replicative drift can strongly affect pathogen diversity within hosts. In our case, bottlenecks and replicative drift appear to be the main drivers of diversity at sites that segregate at the population level.

High-throughput sequencing has revolutionized our ability to measure pathogen variation. It has been used to detect drug resistance [42], to discover novel viruses in nature [43], and to diagnose disease in clinical settings [44]. Our work shows that high-throughput sequencing can also provide important insights into the ecology and evolution of host–pathogen interactions, especially when combined with nested disease models. The increasing availability of both parameterized models [35] and genomic data [45] suggests that our approach of using genetic data to challenge models of nested disease dynamics may be widely applicable.

## Materials and methods

### Ethics statement

This research was conducted under United States Department of Agriculture Animal and Plant Health Inspection Service permit P526P-12-01466.

### Model description

The gypsy moth baculovirus LdMNPV is a double-stranded DNA virus with an approximately 161-kb genome. The virus belongs to the family Baculoviridae, and—like all baculoviruses—it exists in 2 forms, as an "occlusion body" that is highly stable in the environment due to its protective proteinaceous matrix and as a "budded virus" that is released from cells during replication within hosts.

The gypsy moth baculovirus is transmitted when larvae consume occlusion bodies while feeding on foliage [29]. If the resulting virus population grows inside the host to a sufficiently large size, the larva dies, releasing new occlusion bodies onto the foliage. These occlusion bodies are then available to be consumed by additional conspecifics (the virus is species specific [24]), leading to very high infection rates in high-density populations [25]. During the fall and winter, when the insect is in the egg stage, the virus persists in locations where the virus may be protected from degradation by ultraviolet light such as beneath egg masses laid on cadavers [46, 47]. Genetic drift in the gypsy moth baculovirus may therefore be affected by population processes at multiple scales, including within individual hosts and across the host population.

Exposure to the virus results in an initial population of only a few virus particles [15, 48], and the population size in the host remains small for a substantial period of time following exposure [15, 27]. Our model of pathogen growth within hosts therefore tracks population sizes from the initial population bottleneck through the stochastic growth of the pathogen population, until death or recovery. Our model thus explicitly includes genetic drift (Fig 1).

Our within-host model is based on a birth–death model [16], which describes probabilistic changes in population sizes over time. In birth–death models, the probability of a birth or a death in a small period of time increases with the population size [49]. When the population size is small in a birth–death model, it is possible for extinction to occur due to a chance preponderance of deaths over births, even if the per capita birth rate exceeds the per capita death rate. Birth–death models are therefore well suited to describe the demographic stochasticity that underlies replicative drift.

In our within-host birth–death model, pathogen extinction is equivalent to the clearance of the infection by the host. If the pathogen does not go extinct, its population eventually becomes large enough that the effects of stochasticity are negligible [50], leading to host death when the population reaches an upper threshold. In previous work, we showed that linear birth–death models are insufficient to explain data on the speed of kill of the gypsy moth baculovirus, whereas models that allow for nonlinearities due to the immune system produce a better explanation for the data [15].

Our within-host model therefore describes virus removal as the outcome of a process that begins with the insect’s immune system releasing chemicals that activate the phenol-oxidase pathway. This release causes virus particles to be encapsulated and destroyed by host immune cells, and it also incapacitates the immune cell [51, 52]. Our model therefore follows standard predator-prey–type immune system models [53], in which the pathogen is the prey and the immune cells are the predator—except that here, the immune cells do not reproduce over the timescale of a single infection. The pathogen population in the model may then be driven to 0 because of interactions with the host immune system, or it may persist long enough to overwhelm the host immune system, leading to exponential pathogen growth and eventual host death. Which outcome occurs depends on the initial pathogen population size and on demographic stochasticity during the infection.

In our model, the initial pathogen population size within a host is drawn from a Poisson distribution (Fig 1) [15]. If the infecting cadaver is composed of multiple strains, the model draws an initial population size for each strain from a multinomial distribution, such that the probability of sampling a particular strain from the infecting cadaver depends on the frequency of that strain in the cadaver. This process creates a transmission bottleneck. Next, the model tracks the population size of each virus strain over the course of the infection. Changes in the relative frequencies of these strains over time creates replicative drift. The host dies when the total pathogen population size exceeds an upper threshold. The frequency of virus strains at the time of host death determines the frequency of strains in the newly generated cadaver.

We model pathogen dynamics at the scale of the entire host population first by using a stochastic Susceptible-Exposed-Infectious-Removed (SEIR) model to describe epizootics (in our case, the infected *I* class consists of infectious cadavers in the environment, which we symbolize as *P* for pathogen). Our SEIR model is modified to allow hosts to vary in infection risk, an important feature of gypsy moth virus transmission [29, 54], and to allow exposed hosts to be reexposed because infected gypsy moth larvae continue to consume foliage until shortly before death [55]. For computational convenience [56], most SEIR models assume that the time between exposure and infectiousness follows a gamma distribution [35]. We instead allow this time to be determined by our within-host model so that the within-host model is nested inside the stochastic SEIR model. As in the within-host model, the frequency of different virus strains at the population scale can drift due to chance events, such as the exposure of hosts to one cadaver and not another. Our between-host model therefore adds an additional source of drift to our nested models.

Over longer timescales, gypsy moth populations go through host–pathogen population cycles, in which host outbreaks are terminated by baculovirus epizootics. This pattern is typical of many forest-defoliating insects [24]. The resulting predator-prey–type oscillations drive gypsy moth outbreaks at intervals of 5 to 9 years (we neglect the effects of the gypsy moth fungal pathogen *Entomophaga maimaiga*, which had only modest effects in our study areas in Michigan, US, in the years when we collected our samples) [57]. Virus infection rates are very low between insect outbreaks [29], which may strengthen the effects of genetic drift.

Gypsy moths have only 1 generation per year and thus only 1 epizootic per year. We therefore nest our within-host/SEIR-type model into a model that describes host reproduction and virus survival after the epizootic (Fig 1). The SEIR model determines which hosts die during the epizootic and which virus strains killed those hosts. This information is used in difference equations that describe the reproduction of the surviving hosts, the survival of the pathogen over the winter, and the evolution of host resistance, an important factor in gypsy moth outbreak cycles [29, 36].

By explicitly tracking the dynamics of individual hosts and pathogens, our model inherently includes the effects of genetic drift. We also tested whether a simple model of purifying selection or a model of de novo mutation could explain the patterns of diversity in the data without invoking drift within hosts. If these models were to fail to explain the patterns of diversity seen in our data, more complex models of evolution would need to be considered. In the gypsy moth baculovirus system, however, mutation rates are likely low [58, 59], spatial structure appears to be weak [34] (Section A in S1 Text), and evidence of selection acting within hosts is lacking (Section H in S1 Text). Drift therefore seems likely to play a strong role in shaping virus diversity.

To show that the different sources of drift in our model are actually necessary to explain the data, we created 3 alternative models. All 3 alternative models simplify the effects of genetic drift, but 1 also allows for effects of purifying selection. For the first alternative model, we simplified the effects of genetic drift by assuming that the relative frequencies of different virus strains within hosts do not change during pathogen population growth within hosts. To do this, we altered the model output such that the relative frequencies of virus strains released from a host upon host death were equal to the relative frequencies of virus strains just after the transmission bottleneck, thereby eliminating the effects of replicative drift (Fig 1). For the second alternative model, we further simplified the effects of drift by assuming that the relative frequencies of virus strains at the end of an infection were the same as their relative frequencies in the infectious cadaver that initiated the infection, thereby eliminating both replicative drift and transmission bottlenecks (Fig 1). For the third alternative model, we added purifying selection to the second alternative model, which lacked both replicative drift and transmission bottlenecks, by assuming that each host was susceptible to only a random subset of virus strains. Exposure would therefore only result in death if a host was susceptible to one or more virus strains in the cadaver to which it was exposed. The relative frequencies of virus strains released upon death was then equal to the relative frequencies of virus strains to which that host was susceptible in the infecting cadaver.

### Baculovirus sequencing

We collected larvae from outbreaking gypsy moth populations in Michigan between 2000 and 2003 (Section A in S1 Text), and we reared the larvae until they pupated or died of infection [25]. The virus population from each virus-killed larva was passaged once by infecting 75 larvae with liquefied cadaver to generate enough virus for DNA extraction. We then extracted DNA following a standard baculovirus DNA extraction protocol, and we amplified the DNA using whole-genome amplification (REPLI-g UltraFast Mini kit from Qiagen).

We constructed sequencing libraries using the Nextera DNA Sample Prep Kit (Illumina-compatible, #GA0911-96) with custom barcodes to distinguish between the virus communities of different hosts. Our barcodes consisted of the first 96 indexes proposed by reference [60] (Section A in S1 Text). Sequencing was carried out as 2 sets of libraries, run on individual lanes of a HiSeq2000 at the University of Illinois at Urbana–Champaign, producing 100-cycle single-end reads. Samples were separated by barcode using the standard Illumina pipeline, and adaptor contamination was removed using “trim_galore.” Reads were mapped to the first-sequenced gypsy moth baculovirus genome [61] using “bowtie2” [62] with the parameter set “very fast” (Section A in S1 Text). Overall mean coverage was 886x and varied across samples from 202x to 1,497x (Section A in S1 Text). Variant calling was carried out using VarScan version 2.3.9 [63]. More details can be found in Section A of S1 Text.

## Supporting information

S1 Text Supplemental information. (PDF)

S1 Data Observed mean nucleotide diversities. (CSV)

S2 Data Major allele frequencies shown in Fig 4. (CSV)

S3 Data Sample names, collection sites, and collection years. (CSV)

S4 Data Consensus sequences. Columns show data for different loci. Rows show data for different samples. Single dots indicate loci where the consensus sequence matched the reference genome.
(CSV)

S5 Data *F*_*ST*_ values. (CSV)

S6 Data Mean genome coverage for each sample. (CSV)

S7 Data Mean sequencing depth for each locus. (CSV)

S8 Data Percent of reads mapping to LdMNPV in BLAST search. LdMNPV, *Lymantria dispar* multiple nucleopolyhedrovirus.
(CSV)

S9 Data Observed effective number of alleles. (CSV)

S10 Data Observed proportion of polymorphic sites. (CSV)

S11 Data Observed relative nucleotide diversities. (CSV)

S12 Data Major allele frequencies in simulated single-infected host. (CSV)

S13 Data Major allele frequencies in simulated multiple-infected host. (CSV)

## Abbreviations

LdMNPV: *Lymantria dispar* multiple nucleopolyhedrovirus
NCBI: National Center for Biotechnology Information
SEIR: Suspectible-Exposed-Infectious-Removed
SRA: Sequence Read Archive
