# Supplementary material for: Effects of multiple sources of genetic drift on pathogen variation within hosts
Source: PLoS Biol. 2018 Mar 28;16(3):e2004444. doi: 10.1371/journal.pbio.2004444 (PMC5891033; doi:10.1371/journal.pbio.2004444)
Supplement: S1 Text — (PDF) [file pbio.2004444.s001.pdf]

Supplemental Information

A Data Collection, Sequencing, and Bioinformatics

We collected larvae from outbreaking gypsy moth populations in Michigan between 2000 and 2003 (Fig S1, table S1), and we reared them until death or pupation at 26 °C in the lab in individual rearing cups containing an artificial wheat-germ diet [1]. Virus-killed larvae can often be identified visually, but in cases of uncertainty, we examined smears under the microscope for the presence of occlusion bodies, which are large enough to be apparent at 400$\times$ magnification [2]. Virus-killed cadavers were then transferred to 1.5 ml centrifuge tubes where they were stored in distilled water at -20 °C.

We amplified each virus isolate by passaging it through larvae from the New Jersey Standard Strain in the late third and early fourth instars (= developmental stages). As we describe below, we passaged virus through a large number of hosts to avoid introducing a population bottleneck. To amplify the virus, we used fine-tip transfer pipettes to apply four drops of each homogenized sample to three 6 oz. plastic rearing cups. Each cup contained approximately 2 oz. of artificial diet. The virus solution was spread across the diet using plastic-bristle paintbrushes, which were discarded after a single use. Cups were left open to dry for approximately 15 minutes, after which 25 healthy larvae were added to each cup. To confirm that there was no cross contamination between virus cups, we also mock infected control larvae using distilled water. No virus-caused deaths occurred in the controls. Post-infection, larvae were inspected regularly from day 10 to day 18, and intact dead larvae were carefully transferred to 50 ml plastic centrifuge tubes using soft forceps. Following transfers from each cup, the soft forceps were disinfected with 10% bleach solution and wiped to avoid contamination between virus samples. Tubes of passaged virus were stored at 4 °C.

We isolated virus from these samples using the following protocol. First, we shook each tube vigorously to release the virus from the cadavers. Second, we removed intact pieces of host insects by filtering each virus solution through muslin into 1.5 ml centrifuge tubes, after which the muslin was discarded. Third, we centrifuged each tube for 10 minutes at 5000 $\times$ g to pellet the virus, and we discarded the supernatant. Fourth, we added 1 ml of distilled water, we homogenized the solution through mixing on a tabletop vortex, and we repeated the centrifuge step. The pellet was then re-suspended in 500 μl of distilled water and stored at -20 °C.

To extract DNA, we followed a modified version of the protocol of [3]. Briefly, we thawed each virus solution overnight at 4 °C. We then transferred 400 μl of the virus to a new 1.5 ml centrifuge tube, and added 400 μl of a solution of 2% sodium dodecyl sulfate in distilled water. Tubes were inverted repeatedly for 1 minute, and stored overnight at room temperature. The following day, we centrifuged each sample at 5000 $\times$ g for 15 minutes. We then discarded the supernatant, and we re-suspended each pellet in 1 ml of distilled water, before adding 500 μl of an alkaline solution (0.3 M sodium carbonate, 0.03 M EDTA, 0.51 M sodium chloride in

distilled water) to free the virions from the occlusion bodies. We incubated each solution for 1 hour at 37 °C and then centrifuged it at 3000 × g for 5 minutes. Next, we transferred the supernatant to a new centrifuge tube, we centrifuged each tube at 14,000 × g for 30 minutes, and we discarded the supernatant. We then re-suspended each pellet in 200 μl of sterile TE buffer (0.01 M Tris-HCl, 0.001 M EDTA in distilled water) by gently pipetting up and down to break up the pellet. All samples were stored overnight at room temperature. Next, we released DNA from the virions by adding 200 μl of extraction buffer (0.01 M Tris-HCl, 0.001 M EDTA, 0.2% potassium chloride, 0.2% sarkosyl in distilled water), and 4 μl proteinase K. We mixed the tubes by inverting them, and we incubated the solution at 65 °C for 3 hours.

To recover the DNA, we used a phenol-chloroform DNA extraction. Following standard protocol [4], we added 404 μl of 25:24:1 phenol-chloroform isoamyl alcohol to each tube to generate a 1:1 ratio with the sample by volume. We mixed the tubes by gently inverting them for 2 minutes. We centrifuged the samples at 14,000 × g for 20 minutes, and we carefully transferred the top layer to new 1.5 ml centrifuge tubes. Next, we added 480 μl of isopropyl alcohol to the tubes, we mixed them for 2 minutes by gentle inversion, and we placed them on ice for 2 hours. We then centrifuged the samples at 3000 × g for 5 minutes and carefully discarded the supernatant. Next we added 500 μl of 70% ethanol, centrifuged at 3000 × g, and again discarded the supernatant. The samples were left open under a fume hood for 30 minutes to allow the remaining ethanol to evaporate, after which 20 μl of water was added to the samples, and they were stored at -20 °C.

We quantified the amount of DNA in our samples using a spectrophotometer (NanoDrop 2000c). Because our DNA concentrations were low (< 20 pg/μl), we amplified our samples using the whole genome amplification REPLI-g UltraFast Mini kit from Qiagen, following the standard Qiagen protocol. After amplification, DNA concentrations were re-quantified in a spectrophotometer and then normalized to 50 ng/μl. We used the Nextera DNA Sample Prep Kit (Illumina-compatible, # GA0911-96), following the standard protocol for use with custom adaptors, to prepare libraries for Illumina sequencing with custom barcodes. We used the first 96 indexes (table S2) proposed by Meyer and Kircher [5]. After prepping the samples using Nextera, we again quantified DNA concentrations using a spectrophotometer, and we combined the samples into 3 libraries, such that each index was used only once in each library.

Illumina sequencing was performed at the University of Illinois at Urbana-Champaign. This sequencing was carried out as two sets of libraries, run on individual lanes of a HiSeq2000 at 96- and 62-plex respectively, producing 100 cycle single-end reads. Samples were separated according to barcodes using the standard Illumina pipeline. Control, poor coverage, and duplicate libraries were excluded from further analysis, leaving us with 143 unique samples.

Examination of the reads using FastQC
(`http://www.bioinformatics.babraham.ac.uk/projects/fastqc/`)
revealed Nextera adaptor contamination. These contaminated sequences were removed using the wrapper "trim_galore" with default parameters
(`http://www.bioinformatics.babraham.ac.uk/projects/trim_galore/`).
We then mapped the remaining sequences to the *Lymantria dispar* multiple nucleopolyhe-

drovirus (LdMNPV) reference genome [6] using "bowtie2" [7], with parameter set "very-fast". The output "sam" files were converted to "bam" files using "samtools view" [8, 9]. "bam" files were sorted using "samtools sort", and the sorted files were converted to "mpileup" files using "samtools mpileup" with minimum mapping quality 20 and minimum sequence base quality 30. Consensus sequences were then generated and variant calling was simultaneously performed using the function "mpileup2cns" in the program "VarScan" version 2.3.9 [10]. Variant sequences were called at minimum coverage 100. Non-homozygosity was assigned if allele frequencies were between 0.025 and 0.975. The majority allele at every locus was recorded as the consensus sequence for each sample. Repeating our analyses ignoring adaptor contamination, and mapping reads with "bowtie" [11], yielded similar results.

As is often the case, most sites were conserved both within and between our samples (i.e. individual infected hosts). Because conserved sites provide little information, we identified sites that were uniform within samples, but that segregated between at least 7 samples ($\approx 5\%$). This criterion produced the 712 segregating sites that we focus on in the main text. In Fig S1, we show the pairwise similarity between each of our samples at these 712 sites. In Fig S2, we show that the population structure at these 712 sites is low (where populations are defined by the combination of location and year from which a sample was collected).

This pipeline yielded an overall mean genome coverage of 886x per sample, with a range between samples of 202x to 1497x (Fig S3). In Fig S4, we show the average sequencing depth at each site in the genome, and we also show that the segregating sites are spread throughout the virus genome.

We also performed BLAST searches for each of our 143 samples to quantify levels of non-target DNA in our sequence reads. To do this, we converted 10,000 reads from each of our adaptor trimmed FASTQ files into FASTA format. We ran a 'blastn' query for each FASTA file against the 'nt' database using the options 'max_target_seqs 1' and 'max_hsps 1' to ensure reporting of only a single match for each read. We recorded the scientific name and the subject title for each match. In each of our 143 samples, the most common hit was to the gypsy moth virus LdMNPV. Overall, 90.8% of all reads had their best hit classified as "viruses". This percentage varied between samples with a standard deviation of 11.2%. Nevertheless 99.7% of all reads whose best hit was to a virus showed a best match to LdMNPV, suggesting negligible contamination from non-LdMNPV viruses. In approximately two thirds of the remaining samples, the second most common hit was to *Escherichia coli*. To confirm that our estimates of nucleotide diversity were not influenced by DNA contamination in our samples, we performed a linear regression of nucleotide diversity on the fraction of reads that mapped to LdMNPV. We found no significant effect (Fig S5).

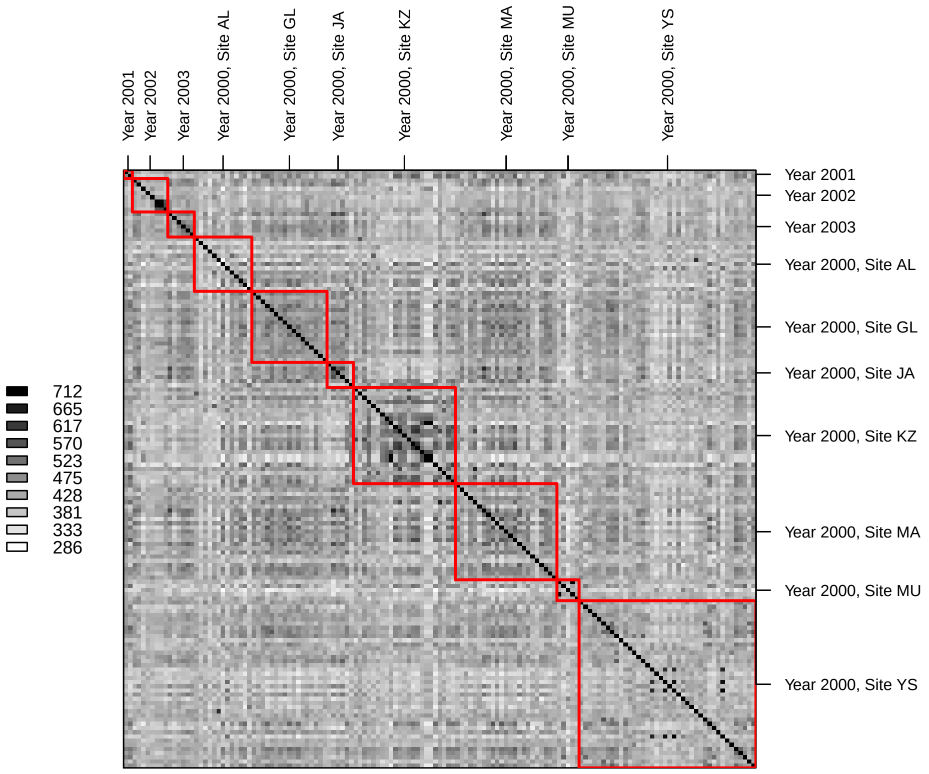

Figure S1: Virus strain collection information and pairwise similarity at segregating sites. Samples were collected from 7 sites over 4 years, although most were collected in 2000. Above, each sample was assigned a column and a row, such that the nth column (read from left to right) represents the same sample as the nth row (read from top to bottom). Red boxes outline samples collected from the same year, or the same year and collection site for samples collected in 2000. The intensity of the shading of each pixel shows the pairwise similarity between consensus sequences at 712 segregating sites used for downstream analysis. The shading within the red boxes is only slightly more intense than the shading outside the red boxes, indicating that spatial structure in this pathogen is weak. Collection site information is provided in table S1. Values underlying this figure can be calculated using data in S3 Data and S4 Data.

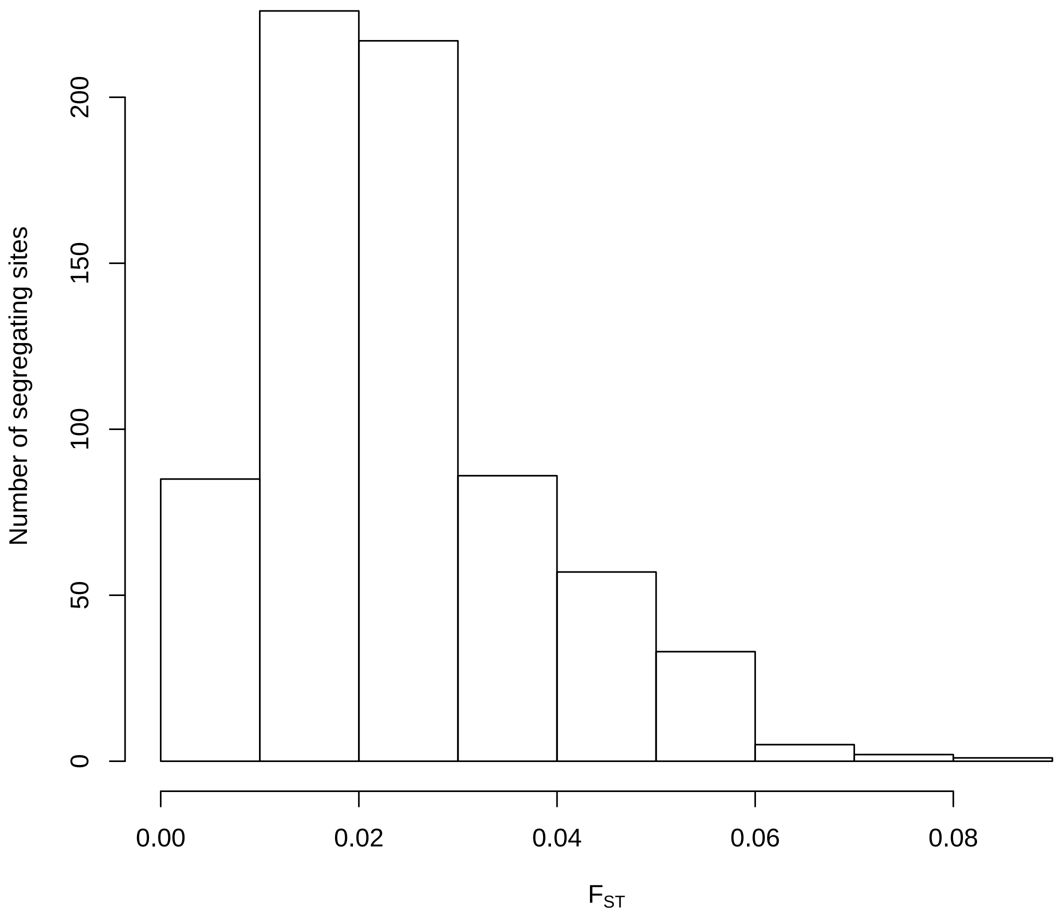

Figure S2: Histogram of F_{ST} values for each of the 712 segregating sites. Populations here are defined as the consensus sequences of samples collected from the same site in the same year. Low F_{ST} values confirm previous work that there is little population structure in the gypsy moth baculovirus across Michigan [12]. Values underlying histogram are provided in S5 Data.

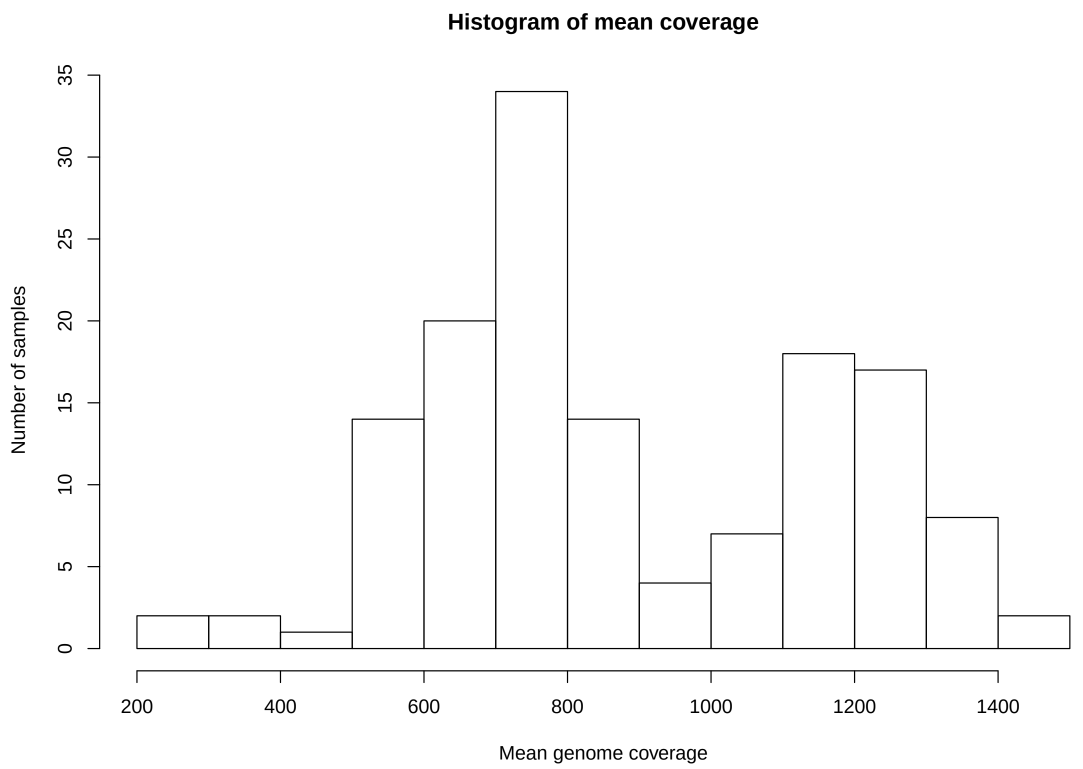

Figure S3: Mean genome coverage for each of the 143 sequenced samples. Values underlying histogram are provided in S6 Data.

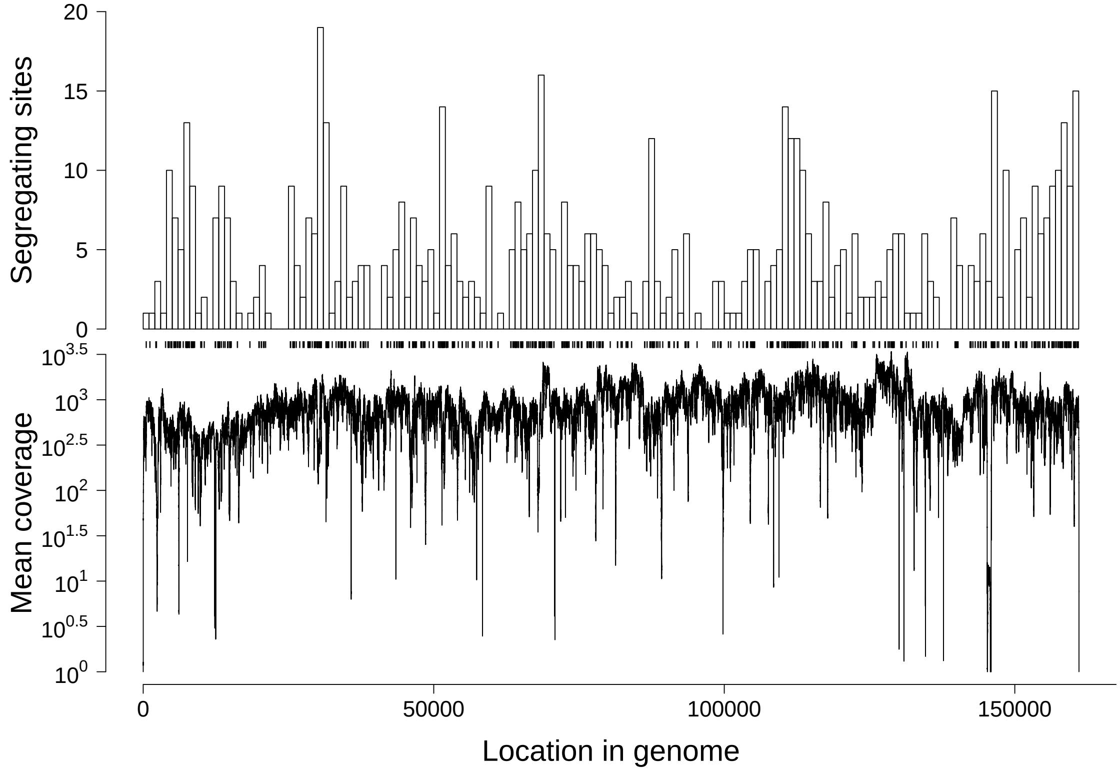

Figure S4: Location of segregating sites, and mean sequencing depth across the genome. The top panel shows the location of segregating sites across the genome, with locations corresponding to GenBank accession number NC_001973.1 [6]. Each segregating site is marked by a vertical slash in the middle of the figure. The bottom panel shows the mean sequencing depth at each site in the genome. Instead of being restricted to sites of particularly high or particularly low sequencing depth, segregating sites are spread throughout the virus genome. Plotted values are provided in S7 Data.

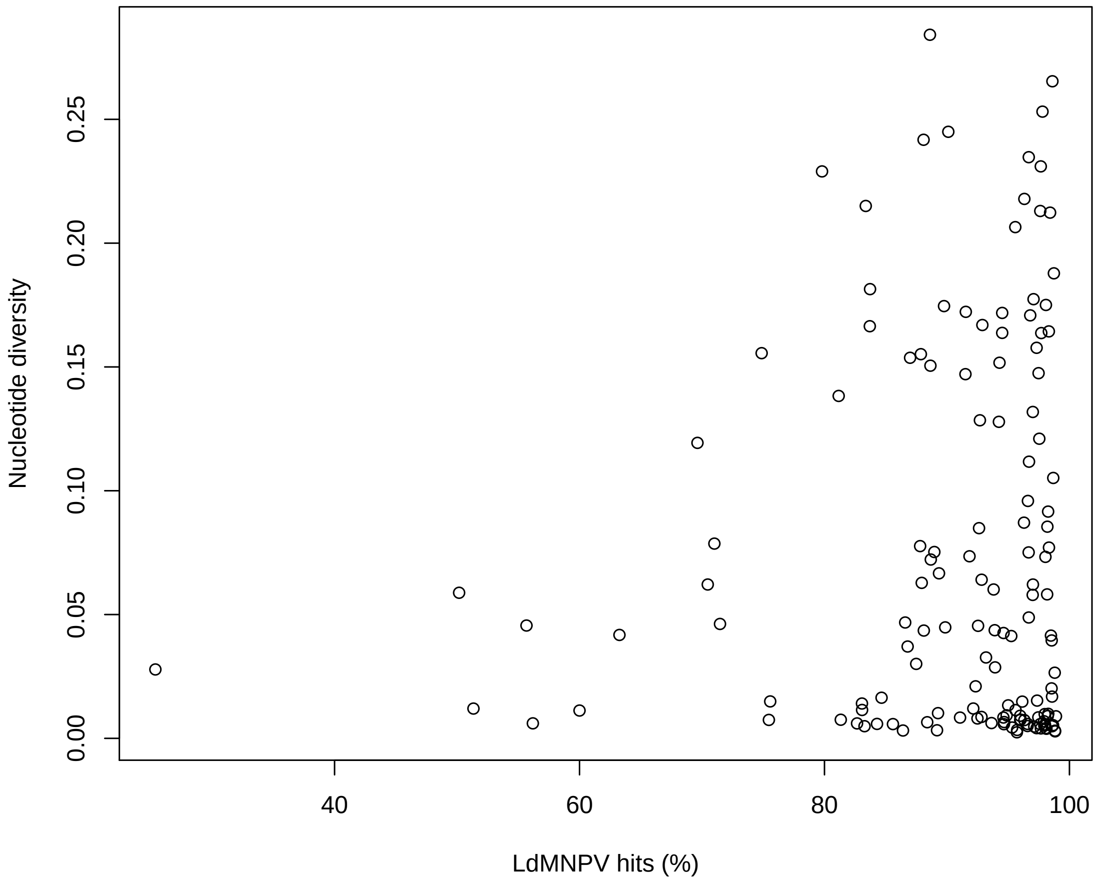

Figure S5: Nucleotide diversity versus the percentage of sequence reads with a best hit to LdMNPV. The lack of correlation between these variables suggests that our conclusions are unlikely to have been affected by the presence of non-target DNA. Plotted values are provided in S8 Data.

Table S1: Collection site information. All sites are in Michigan, USA. Latitudes and longitudes were estimated using Google Maps, by the recollections of the second author and his field assistant of last resort, Dr. Alison F. Hunter.

**Collection site**	**Nearest metropolitan area**	**Latitude**	**Longitude**
AL	Allegan	42.53	-85.88
GL	Gladwin	43.99	-84.40
JA	Jackson	42.27	-84.36
KZ	Kalmazoo	42.37	-85.52
MA	Manistee	44.23	-86.04
MU	Muskegon	43.27	-86.12
YS	Yankee Springs	42.63	-85.45

Table S2: Illumina barcodes. Adaptors were designed using the custom barcode template provided by the Illumina-compatible Nextera sample prep kit. Barcode sequences themselves were taken from [5].

Index ID	Adaptor 2 sequence
INDEX-1	CAAGCAGAAGACGGCATACGAGATCCTGCGACGGTCTGCCTTGCCAGCCCGCTCAG
INDEX-2	CAAGCAGAAGACGGCATACGAGATTGCAGAGCGGTCTGCCTTGCCAGCCCGCTCAG
INDEX-3	CAAGCAGAAGACGGCATACGAGATACCTAGGCGGTCTGCCTTGCCAGCCCGCTCAG
INDEX-4	CAAGCAGAAGACGGCATACGAGATTTGATCCCGGTCTGCCTTGCCAGCCCGCTCAG
INDEX-5	CAAGCAGAAGACGGCATACGAGATATCTTGCCGGTCTGCCTTGCCAGCCCGCTCAG
INDEX-6	CAAGCAGAAGACGGCATACGAGATTCTCCATCGGTCTGCCTTGCCAGCCCGCTCAG
INDEX-7	CAAGCAGAAGACGGCATACGAGATCATCGAGCGGTCTGCCTTGCCAGCCCGCTCAG
INDEX-8	CAAGCAGAAGACGGCATACGAGATTTCGAGCCGGTCTGCCTTGCCAGCCCGCTCAG
INDEX-9	CAAGCAGAAGACGGCATACGAGATAGTTGGTCGGTCTGCCTTGCCAGCCCGCTCAG
INDEX-10	CAAGCAGAAGACGGCATACGAGATGTACCGGCGGTCTGCCTTGCCAGCCCGCTCAG
INDEX-11	CAAGCAGAAGACGGCATACGAGATCGGAGTTCGGTCTGCCTTGCCAGCCCGCTCAG
INDEX-12	CAAGCAGAAGACGGCATACGAGATACTTCAACGGTCTGCCTTGCCAGCCCGCTCAG
INDEX-13	CAAGCAGAAGACGGCATACGAGATTGATAGTCGGTCTGCCTTGCCAGCCCGCTCAG
INDEX-14	CAAGCAGAAGACGGCATACGAGATGATCCAACGGTCTGCCTTGCCAGCCCGCTCAG
INDEX-15	CAAGCAGAAGACGGCATACGAGATCAGGTCGCGGTCTGCCTTGCCAGCCCGCTCAG
INDEX-16	CAAGCAGAAGACGGCATACGAGATCGCATTACGGTCTGCCTTGCCAGCCCGCTCAG
INDEX-17	CAAGCAGAAGACGGCATACGAGATGGTACCTCGGTCTGCCTTGCCAGCCCGCTCAG
INDEX-18	CAAGCAGAAGACGGCATACGAGATGGACGCACGGTCTGCCTTGCCAGCCCGCTCAG
INDEX-19	CAAGCAGAAGACGGCATACGAGATGAGATTCCGGTCTGCCTTGCCAGCCCGCTCAG
INDEX-20	CAAGCAGAAGACGGCATACGAGATGAGCATGCGGTCTGCCTTGCCAGCCCGCTCAG
INDEX-21	CAAGCAGAAGACGGCATACGAGATGTTGCGTCGGTCTGCCTTGCCAGCCCGCTCAG
INDEX-22	CAAGCAGAAGACGGCATACGAGATCCAATGCCGGTCTGCCTTGCCAGCCCGCTCAG
INDEX-23	CAAGCAGAAGACGGCATACGAGATCGAGATCCGGTCTGCCTTGCCAGCCCGCTCAG
INDEX-24	CAAGCAGAAGACGGCATACGAGATCATATTGCGGTCTGCCTTGCCAGCCCGCTCAG
INDEX-25	CAAGCAGAAGACGGCATACGAGATGACGTCACGGTCTGCCTTGCCAGCCCGCTCAG
INDEX-26	CAAGCAGAAGACGGCATACGAGATTGGCATCCGGTCTGCCTTGCCAGCCCGCTCAG

Continued on Next Page…

Table S2 – Continued

Index ID	Adaptor 2 sequence
INDEX-27	CAAGCAGAAGACGGCATACGAGATGTAATTGCGGTCTGCCTTGCCAGCCCGCTCAG
INDEX-28	CAAGCAGAAGACGGCATACGAGATCCTATCTCGGTCTGCCTTGCCAGCCCGCTCAG
INDEX-29	CAAGCAGAAGACGGCATACGAGATCAATCGGCGGTCTGCCTTGCCAGCCCGCTCAG
INDEX-30	CAAGCAGAAGACGGCATACGAGATGCGGCATCGGTCTGCCTTGCCAGCCCGCTCAG
INDEX-31	CAAGCAGAAGACGGCATACGAGATAGTACTGCGGTCTGCCTTGCCAGCCCGCTCAG
INDEX-32	CAAGCAGAAGACGGCATACGAGATTACTATTCGGTCTGCCTTGCCAGCCCGCTCAG
INDEX-33	CAAGCAGAAGACGGCATACGAGATCCGGATGCGGTCTGCCTTGCCAGCCCGCTCAG
INDEX-34	CAAGCAGAAGACGGCATACGAGATACCATGACGGTCTGCCTTGCCAGCCCGCTCAG
INDEX-35	CAAGCAGAAGACGGCATACGAGATCGGTTCTCGGTCTGCCTTGCCAGCCCGCTCAG
INDEX-36	CAAGCAGAAGACGGCATACGAGATTATTCCACGGTCTGCCTTGCCAGCCCGCTCAG
INDEX-37	CAAGCAGAAGACGGCATACGAGATCCTCCTGCGGTCTGCCTTGCCAGCCCGCTCAG
INDEX-38	CAAGCAGAAGACGGCATACGAGATAGGTATTCGGTCTGCCTTGCCAGCCCGCTCAG
INDEX-39	CAAGCAGAAGACGGCATACGAGATGCATTCGCGGTCTGCCTTGCCAGCCCGCTCAG
INDEX-40	CAAGCAGAAGACGGCATACGAGATTTGCGAACGGTCTGCCTTGCCAGCCCGCTCAG
INDEX-41	CAAGCAGAAGACGGCATACGAGATTTGAATTCGGTCTGCCTTGCCAGCCCGCTCAG
INDEX-42	CAAGCAGAAGACGGCATACGAGATCTGCGCGCGGTCTGCCTTGCCAGCCCGCTCAG
INDEX-43	CAAGCAGAAGACGGCATACGAGATAGACCTTCGGTCTGCCTTGCCAGCCCGCTCAG
INDEX-44	CAAGCAGAAGACGGCATACGAGATGTCCAGTCGGTCTGCCTTGCCAGCCCGCTCAG
INDEX-45	CAAGCAGAAGACGGCATACGAGATACCTGCTCGGTCTGCCTTGCCAGCCCGCTCAG
INDEX-46	CAAGCAGAAGACGGCATACGAGATCCGGTACCGGTCTGCCTTGCCAGCCCGCTCAG
INDEX-47	CAAGCAGAAGACGGCATACGAGATCTTGACCCGGTCTGCCTTGCCAGCCCGCTCAG
INDEX-48	CAAGCAGAAGACGGCATACGAGATCATCATTCGGTCTGCCTTGCCAGCCCGCTCAG
INDEX-49	CAAGCAGAAGACGGCATACGAGATTCTGACTCGGTCTGCCTTGCCAGCCCGCTCAG
INDEX-50	CAAGCAGAAGACGGCATACGAGATTCTAGTTCGGTCTGCCTTGCCAGCCCGCTCAG
INDEX-51	CAAGCAGAAGACGGCATACGAGATGCCATAGCGGTCTGCCTTGCCAGCCCGCTCAG
INDEX-52	CAAGCAGAAGACGGCATACGAGATACCGTCGCGGTCTGCCTTGCCAGCCCGCTCAG
INDEX-53	CAAGCAGAAGACGGCATACGAGATCTTGGTTCGGTCTGCCTTGCCAGCCCGCTCAG

Continued on Next Page…

Table S2 – Continued

Index ID	Adaptor 2 sequence
INDEX-54	CAAGCAGAAGACGGCATACGAGATTACGCCGCGGTCTGCCTTGCCAGCCCGCTCAG
INDEX-55	CAAGCAGAAGACGGCATACGAGATGGACTGCCGGTCTGCCTTGCCAGCCCGCTCAG
INDEX-56	CAAGCAGAAGACGGCATACGAGATGCGCGAGCGGTCTGCCTTGCCAGCCCGCTCAG
INDEX-57	CAAGCAGAAGACGGCATACGAGATGTCGCAGCGGTCTGCCTTGCCAGCCCGCTCAG
INDEX-58	CAAGCAGAAGACGGCATACGAGATCATACGTCGGTCTGCCTTGCCAGCCCGCTCAG
INDEX-59	CAAGCAGAAGACGGCATACGAGATTCAGTATCGGTCTGCCTTGCCAGCCCGCTCAG
INDEX-60	CAAGCAGAAGACGGCATACGAGATCTAAGTACGGTCTGCCTTGCCAGCCCGCTCAG
INDEX-61	CAAGCAGAAGACGGCATACGAGATTTAGCTTCGGTCTGCCTTGCCAGCCCGCTCAG
INDEX-62	CAAGCAGAAGACGGCATACGAGATCGCCGTCCGGTCTGCCTTGCCAGCCCGCTCAG
INDEX-63	CAAGCAGAAGACGGCATACGAGATGTCTTCTCGGTCTGCCTTGCCAGCCCGCTCAG
INDEX-64	CAAGCAGAAGACGGCATACGAGATGCCGGACCGGTCTGCCTTGCCAGCCCGCTCAG
INDEX-65	CAAGCAGAAGACGGCATACGAGATAAGCTGACGGTCTGCCTTGCCAGCCCGCTCAG
INDEX-66	CAAGCAGAAGACGGCATACGAGATGCGCTCTCGGTCTGCCTTGCCAGCCCGCTCAG
INDEX-67	CAAGCAGAAGACGGCATACGAGATCGTAGGCCGGTCTGCCTTGCCAGCCCGCTCAG
INDEX-68	CAAGCAGAAGACGGCATACGAGATATGATTACGGTCTGCCTTGCCAGCCCGCTCAG
INDEX-69	CAAGCAGAAGACGGCATACGAGATGCAGGTTCGGTCTGCCTTGCCAGCCCGCTCAG
INDEX-70	CAAGCAGAAGACGGCATACGAGATAATCGTCCGGTCTGCCTTGCCAGCCCGCTCAG
INDEX-71	CAAGCAGAAGACGGCATACGAGATCGGCCTACGGTCTGCCTTGCCAGCCCGCTCAG
INDEX-72	CAAGCAGAAGACGGCATACGAGATCTATGCCCGGTCTGCCTTGCCAGCCCGCTCAG
INDEX-73	CAAGCAGAAGACGGCATACGAGATGGTTGAACGGTCTGCCTTGCCAGCCCGCTCAG
INDEX-74	CAAGCAGAAGACGGCATACGAGATGAGTTAACGGTCTGCCTTGCCAGCCCGCTCAG
INDEX-75	CAAGCAGAAGACGGCATACGAGATTAGACTACGGTCTGCCTTGCCAGCCCGCTCAG
INDEX-76	CAAGCAGAAGACGGCATACGAGATTCATGCACGGTCTGCCTTGCCAGCCCGCTCAG
INDEX-77	CAAGCAGAAGACGGCATACGAGATGCTTATTCGGTCTGCCTTGCCAGCCCGCTCAG
INDEX-78	CAAGCAGAAGACGGCATACGAGATCAAGGCTCGGTCTGCCTTGCCAGCCCGCTCAG
INDEX-79	CAAGCAGAAGACGGCATACGAGATAGGTTGGCGGTCTGCCTTGCCAGCCCGCTCAG
INDEX-80	CAAGCAGAAGACGGCATACGAGATCTTCTGCCGGTCTGCCTTGCCAGCCCGCTCAG

Continued on Next Page…

Table S2 – Continued

**Index ID**	**Adaptor 2 sequence**
INDEX-81	CAAGCAGAAGACGGCATACGAGATTAATTCTCGGTCTGCCTTGCCAGCCCGCTCAG
INDEX-82	CAAGCAGAAGACGGCATACGAGATGATGCTGCGGTCTGCCTTGCCAGCCCGCTCAG
INDEX-83	CAAGCAGAAGACGGCATACGAGATCCTAGAACGGTCTGCCTTGCCAGCCCGCTCAG
INDEX-84	CAAGCAGAAGACGGCATACGAGATCTAGAGGCGGTCTGCCTTGCCAGCCCGCTCAG
INDEX-85	CAAGCAGAAGACGGCATACGAGATTATCCGGCGGTCTGCCTTGCCAGCCCGCTCAG
INDEX-86	CAAGCAGAAGACGGCATACGAGATAGGCGGCCGGTCTGCCTTGCCAGCCCGCTCAG
INDEX-87	CAAGCAGAAGACGGCATACGAGATGGTCGTTCGGTCTGCCTTGCCAGCCCGCTCAG
INDEX-88	CAAGCAGAAGACGGCATACGAGATCCGCTGGCGGTCTGCCTTGCCAGCCCGCTCAG
INDEX-89	CAAGCAGAAGACGGCATACGAGATGGAACTACGGTCTGCCTTGCCAGCCCGCTCAG
INDEX-90	CAAGCAGAAGACGGCATACGAGATATTGCCACGGTCTGCCTTGCCAGCCCGCTCAG
INDEX-91	CAAGCAGAAGACGGCATACGAGATATATACGCGGTCTGCCTTGCCAGCCCGCTCAG
INDEX-92	CAAGCAGAAGACGGCATACGAGATGATTAGCCGGTCTGCCTTGCCAGCCCGCTCAG
INDEX-93	CAAGCAGAAGACGGCATACGAGATAGAAGTCCGGTCTGCCTTGCCAGCCCGCTCAG
INDEX-94	CAAGCAGAAGACGGCATACGAGATATAGTACCGGTCTGCCTTGCCAGCCCGCTCAG
INDEX-95	CAAGCAGAAGACGGCATACGAGATGATCTCGCGGTCTGCCTTGCCAGCCCGCTCAG
INDEX-96	CAAGCAGAAGACGGCATACGAGATGGCTGCGCGGTCTGCCTTGCCAGCCCGCTCAG

B Calculating Nucleotide Diversity

To quantify pathogen diversity within individual hosts, we used average nucleotide diversity π, a standard population-genetic statistic used to summarize diversity [13]. In Supplemental Information I, we show that similar results are obtained when using alternative summary statistics such as the effective number of alleles A_e, the fraction of polymorphic loci P, and the relative nucleotide diversity $\hat{\pi}$. Nucleotide diversity is the probability that any two randomly selected alleles at a particular site in a population would differ if the population were in Hardy-Weinberg equilibrium. It is defined as:

$$\pi = 1 - \frac{1}{n}\sum_{j=1}^{n}\sum_{i=1}^{k_j} x_{ij}^2. \tag{1}$$

Here π is the nucleotide diversity, k_j is the total number of allelic variants in the population at site j, x_{ij} is the frequency of allele i at site j, and n is the total number of focal sites. In our case, n is equal to the 712 segregating sites identified in Supplemental Information A.

Fig S6 shows nucleotide diversity calculated across sites that did not segregate at the population level, demonstrating that nucleotide diversity is negligible at sites not segregating at the population level. Comparison to Fig 3 in the main text strongly suggests that the pathogen diversity that we observed within hosts was due to exposures to multiple pathogen genotypes, and not to mutation or diversifying selection within hosts.

This is not to say, however, that all variation present within hosts can be explained by coinfection, just that a large fraction of it can be. Because segregating sites comprise 0.4% of the genome, the mean nucleotide diversity across the entire genome can be calculated as $\pi_G = 0.996\pi_{NS} + 0.004\pi_S$, where π_G is the mean nucleotide diversity calculated across the entire genome, π_{NS} is the nucleotide diversity at non-segregating sites and π_S is the nucleotide diversity at segregating sites.

The fraction of overall variation explained by segregating sites is therefore $0.004\pi_S/\pi_G$. In the main text, we report the mean values $\pi_{NS} = 0.001$ and $\pi_S = 0.07$. Accordingly, approximately 23% of the variation can be explained by just the 712 segregating sites. In Fig S7, we show this estimate for each of the 143 samples used in our study. Due to the way nucleotide diversity is calculated, the fraction of variation attributable to segregating sites could only increase if we were more liberal in what we considered to be segregating sites (currently that threshold is somewhat strict, based on a frequency of alternative alleles above about 5% across consensus sequences), and so we are probably underestimating the fraction of variation attributable to coinfection.

In addition, our estimate of nucleotide diversity at non-segregating sites is likely a strong overestimate. This overestimate arises because all next generation sequencing platforms, including the Illumina platform that we used, have high sequencing error rates, and such errors inflate estimates of nucleotide diversity. The low diversity levels at non-segregating sites in our samples (i.e. 0.001) are well within the plausible range for variation generated by sequencing errors alone.

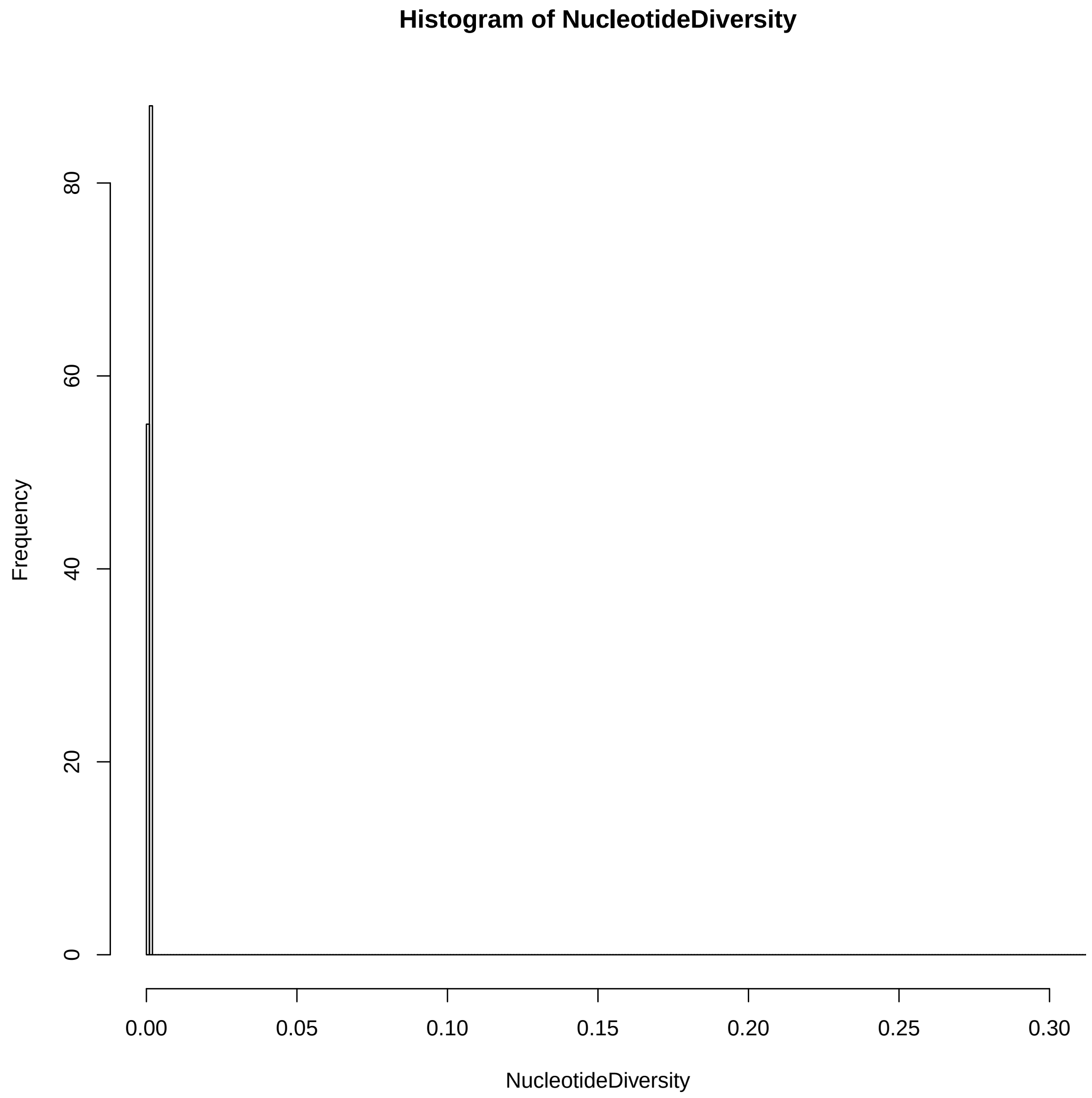

Figure S6: Mean nucleotide diversity at sites that are not segregating at the population level. Values underlying this figure can be calculated using data in S4 Data.

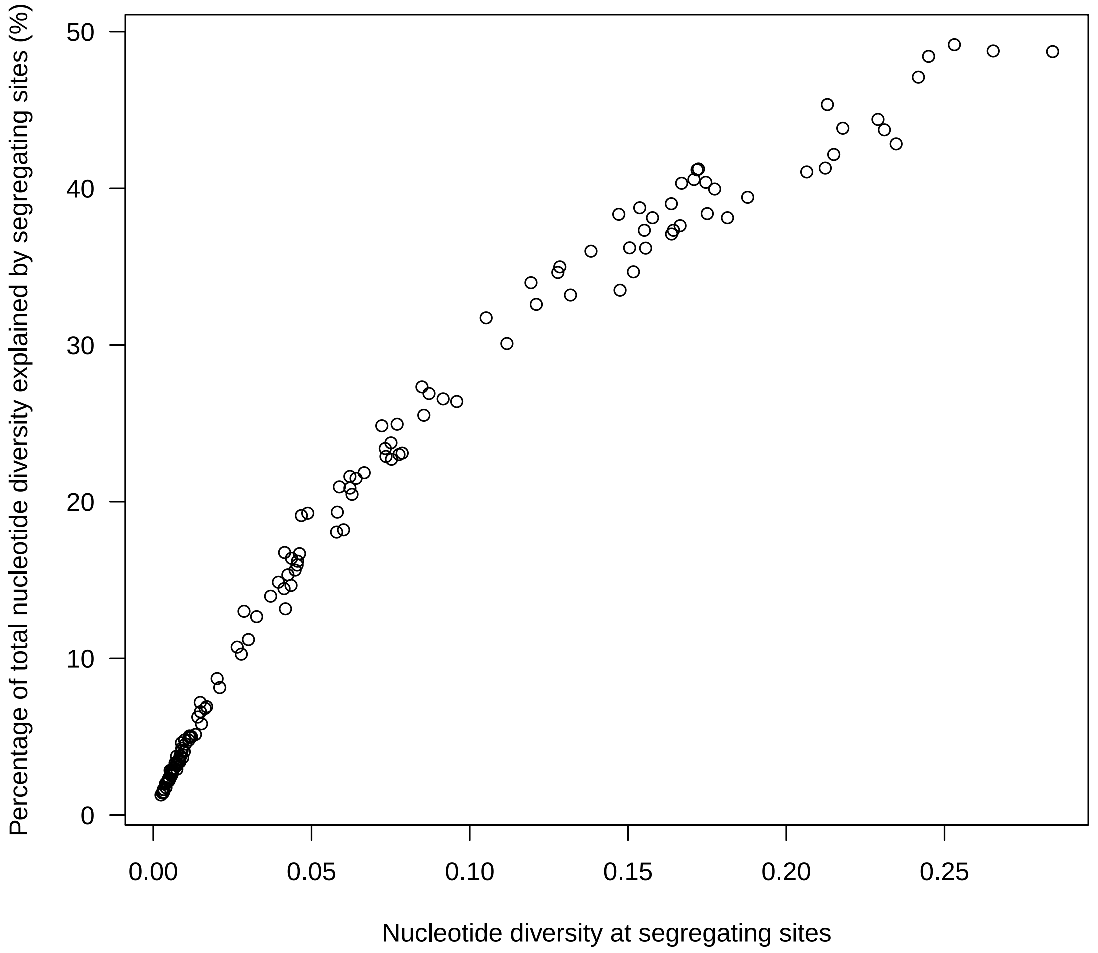

Figure S7: Percent of total nucleotide diversity explained by segregating sites. Note that total nucleotide diversity is probably overestimated due to sequencing error, and so the estimates of variation explained by segregating sites are likely conservative. Values underlying this figure can be calculated using data in S4 Data and S8 Data.

C Nested Model Structure

Our models are nonlinear generalizations of linear birth-death models [14], and so we begin the explanation of the models by first presenting a linear birth-death model. A birth-death model describes probabilistic changes in the size of a population over time, such that the probability of a birth or a death in a small period of time depends on the population size, and only integer population sizes are possible. In the linear case, the model is [15]:

$$P(x_{t+\Delta t} = x_t + 1 | x_t) = r x_t \Delta t + o(\Delta t), \quad (2)$$

$$P(x_{t+\Delta t} = x_t - 1 | x_t) = \alpha x_t \Delta t + o(\Delta t), \quad (3)$$

$$P(x_{t+\Delta t} = x_t | x_t) = 1 - (r + \alpha) x_t \Delta t + o(\Delta t). \quad (4)$$

Here x is the population size, so that the first equation describes the probability of a birth occurring in the time interval $(t, t + \Delta t]$. Because each individual has the same probability of giving birth, the probability of a birth depends on the replication rate r, the number of individuals x_t, and a term $o(\Delta t)$ that describes the probability that multiple events occur in a single time interval Δt. This latter term is assumed to go to zero very rapidly as Δt becomes small ($\lim_{\Delta t \to 0} \frac{o(\Delta t)}{\Delta t} = 0$). The second equation describes the probability of a death occurring in the time interval $(t, t + \Delta t]$, which similarly depends on the death rate α, the number of individuals x_t, and the probability that multiple events occur in a single time interval, which again goes to zero rapidly with Δt. The third equation describes the probability that neither a birth nor a death occurs in $(t, t + \Delta t]$. Because $o(\Delta t)$ goes to zero with Δt, the probabilities sum to 1 as Δt goes to zero [15]. In practice, our models include processes that are not found in the linear birth-death model, notably the response of the immune system, but the linear model nevertheless provides a useful introduction to the overall approach of using birth-death models to represent within-host pathogen population growth.

In birth-death models, the pathogen population is founded by the invasion of a discrete number of pathogen particles into the host. In the linear birth-death model in particular, each of these particles has a constant probability of reproducing or dying, and the resulting births and deaths lead to changes in the pathogen population size within the host. If the pathogen population size reaches 0, which might occur by chance if the initial population size is small, the host recovers, but if the pathogen population instead reaches an upper threshold, the host becomes ill. We can thus use the linear birth-death model to describe both the probability of host illness given exposure to the pathogen, and the incubation time, meaning the time between exposure and illness, as long as we specify the threshold at which symptoms occur [16]. As Fig S8 then shows, the model predicts that there will be variability in incubation times, and in the probability of illness, even if hosts are identical, simply because of the stochasticity inherent in the birth-death process. Note that most of the variability in outcomes is due to events that occur shortly after infection, when pathogen population sizes are small.

In previous work with a colleague [17, 18], we extended the linear birth-death model to include the nonlinearities inherent in the insect immune response, thereby producing a model that provides a more realistic description of within-host baculovirus growth. In this model,

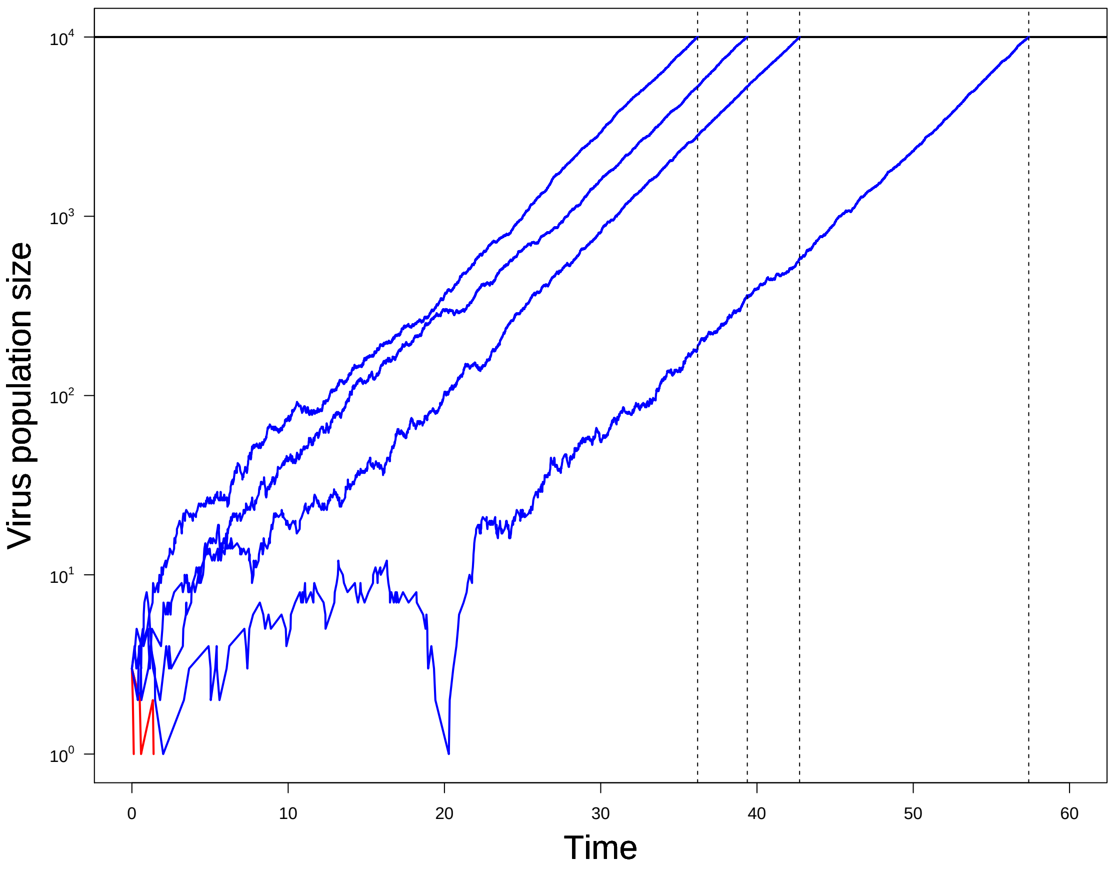

Figure S8: Realizations of the linear birth death model (eqs. 2-4). The solid horizontal line is the threshold pathogen population size at which the host dies, and the dashed vertical lines show times of host deaths. Blue lines show realizations in which the host ultimately dies of the infection, and red lines show realizations in which the host recovers and the pathogen goes extinct. Note that when pathogen populations are small, trajectories are highly variable, which in turn creates variation in the time at which pathogen populations reach the size at which hosts die. Parameters: initial pathogen population size $x_0 = 3$, pathogen replication rate $r = 0.7$ per hour, pathogen death rate $\alpha = 0.5$ per hour, threshold for death $C = 10^4$ pathogen particles.

after a few pathogen particles colonize the host, the particles may again reproduce, as in the linear model, but they may also be bound and destroyed by host immune cells. In insects and other invertebrates, immune cells release chemicals that activate the phenol-oxidase pathway, ultimately resulting in the encapsulation and destruction of pathogen particles [19, 20]. The pathogen population may therefore fall to zero because of interactions with the host immune system, in which case the host recovers from infection, but the immune system may also be overwhelmed by the pathogen, leading to runaway pathogen growth and host death. Accordingly, instead of the linear model probabilities in eqs. (2)-(4), we have:

$$P(x_{t+\Delta t} = x_t + 1, y_{t+\Delta t} = y_t | x_t, y_t) = r x_t \Delta t - o(\Delta t), \quad (5)$$

$$P(x_{t+\Delta t} = x_t - 1, y_{t+\Delta t} = y_t - 1 | x_t, y_t) = \beta x_t y_t \Delta t - o(\Delta t), \quad (6)$$

$$P(x_{t+\Delta t} = x_t, y_{t+\Delta t} = y_t | x_t, y_t) = 1 - r x_t \Delta t - \beta x_t y_t \Delta t - o(\Delta t). \quad (7)$$

Here, x_t and y_t are the respective population sizes of virus particles and immune cells at time t, r is the virus replication rate, and β is the rate at which immune cells encounter and encapsulate pathogen particles. We extend this model, without changing the dynamics in any way, by replacing x_t with $\sum_{i=1}^{x_0} x_{t,i}$ where x_0 is the initial number of virus particles (*not* the number of unique virus strains) that founded the infection, and $x_{t,i}$ denotes the population size resulting from founder virion i at time t. Note the distinction between x_t, the total virus population size at time t, and $x_{t,i}$, the population size of virus particles resulting from virus founder i at time t. The population size resulting from each founder virion can then be explicitly tracked by rewriting eq. (5) as a set of x_0 equations, where x_t is replaced with $x_{t,i}$. We set $x_{0,i}$ equal to 1 for all i, such that each of these x_0 equations describes the population size resulting from a different founder. A crucial point is that multiple founder virus particles may be identical in terms of virus strain identity. To determine the fraction of a cadaver that is comprised of any particular virus strain therefore requires first determining which of the x_0 virus lineages match the strain of interest. We similarly expand eq. (6). This formulation of the model allows us to explicitly capture changes in the virus population composition that are due to replicative genetic drift.

The model defined by the above probabilities describes the stochastic population growth that underlies the genetic drift of pathogen populations inside their hosts. To allow for transmission bottlenecks, we include a submodel that describes the reduction in the pathogen population size that occurs at transmission. In the gypsy moth baculovirus, for example, an infected cadaver releases on the order of 10^9 infectious occlusion bodies [21], but the number of virus particles that successfully invade a host is only 10–100, reflecting a transmission bottleneck [17]. We therefore assume that the initial number of pathogen particles follows a Poisson distribution, according to;

$$x_0 \sim \text{Poisson}\left(\frac{c_1 D}{c_2 + D}\right), \quad (8)$$

Here x_0 is again the initial number of virus particles that colonize a host. To allow for saturation of pathogen invasion sites, the mean of this distribution is calculated from a Michaelis-Menten

function, such that the mean number of colonizing particles is a saturating function of the dose D, with parameters c_1 and c_2. By defining the initial number of immune cells $y_0 \equiv m$, and specifying the virus population size C at which host death occurs, we have fully described the dynamics of our within-host model. To explicitly model genetic drift due to transmission bottlenecks, we randomly sample x_0 virus genomes with replacement from the population of virions that comprises the infectious cadaver to which a host was exposed. Specifically, virus strains are sampled using a multinomial distribution, in which the probability of sampling a particular strain depends on its frequency in the infectious cadaver. This produces x_0 virus particles that can then be tracked using eqs. (5)-(7). Reinfections are treated in the same way as primary infections. If multiple host exposures occur, the host dies at the time that the total number of particles first exceeds the host-death threshold. The frequency of each virus strain at the time of host death then becomes the frequency of each strain in the newly generated cadaver. We allowed for 50 unique virus genotypes, but using larger numbers of strains had negligible effects on our results.

The above text describes the model that includes drift due to both transmission bottlenecks and replicative genetic drift. To remove replicative genetic drift, we again used eqs. (5)-(7) to determine the time of death, but we no longer tracked the identity of each individual virus particle during growth within hosts. The time to death is therefore the same, but the composition of the virus population is unaffected by stochasticity during growth within hosts. To eliminate transmission bottlenecks, we removed the sampling process during virus colonization, such that the virus community infecting a host was identical in composition to the community to which the host was exposed. Finally, to add purifying selection, we allowed hosts to be resistant to a subset of virus strains. The three alternative models are described in more detail later in this section.

We tracked a fixed number of virus strains (i.e. 50) that changed in relative and absolute frequency over time, but otherwise did not change. These simulated strains varied at 712 loci, to match the variability in our sequence data (further described in Supplemental Information F). We therefore did not include mutation or recombination in any of the models presented in the main text. These simplifications are justified by the biology of the system, and they allowed us to greatly improve the tractability of the system, by tracking a fixed number of strains (i.e. 50). Although there are no estimates of baculovirus mutation rates, data from other double stranded DNA viruses suggests that mutation rates are likely to be on the order of 10^{-7} substitutions per locus per infected cell [22]. In Supplemental Information E, we show that this mutation rate generates too little variation to explain our data, and that in fact, no mutation rate can simultaneously explain both the high and low diversity infections seen in our data. We also are unaware of any estimates of baculovirus recombination rates, but given that recombination is not a necessary part of the baculovirus lifecycle, the frequency of recombination is probably small over ecological timescales.

Realizations of our within-host model show that most of the variability in the time at death is due to events that occur early on in the infection (Fig S9), as in the linear birth-death model (Fig S8). Comparison of Fig S9 and Fig S8, however, makes clear that allowing for the non-

linearities inherent in the immune system slows the growth of the virus population early in the infection. Because the immune system thus keeps pathogen population sizes low for longer, it strengthens the effects of drift.

The nonlinear birth-death model is used once a host becomes infected, to determine whether the host will die of the infection, and if so, when death will occur. To determine whether a host becomes infected in the first place, we use a stochastic SEIR model, modified to allow for host variation in infection risk [23,24], and to allow the birth-death model to determine the time until death, and the probability of death. In our selection model, a host only dies if the cadaver that initiated the infection also contains at least one virus strain to which that the host is susceptible. Allowing our birth-death model to determine virus dynamics within hosts is crucial, because the within host dynamics drive replicative drift. The resulting distribution of times to death is nevertheless roughly similar to a gamma distribution [17], which determines the time to death in standard SEIR models. It is therefore worth observing that, if incubation times within hosts did follow a gamma distribution, then the deterministic equivalent of our stochastic SEIR model would be:

$$\frac{dS}{dt} = -\bar{\nu} S P \left[\frac{S(t)}{S(0)}\right]^{V}, \tag{9}$$

$$\frac{dE_1}{dt} = \bar{\nu} S P \left[\frac{S(t)}{S(0)}\right]^{V} - k\delta E_1, \tag{10}$$

$$\frac{dE_i}{dt} = k\delta E_{i-1} - k\delta E_i, \text{ for } i = 2, \ldots k, \tag{11}$$

$$\frac{dP}{dt} = k\delta E_k - \mu P. \tag{12}$$

Here, S and P are the densities of healthy hosts and infectious cadavers, respectively, while E_i is the density of exposed but not yet infectious hosts in exposure class i. Allowing for k exposed classes produces a gamma distribution of times to death, with mean $1/\delta$ and coefficient of variation (C.V.) $1/\sqrt{k}$ [25]. Previous work has shown that infection risk varies greatly across individuals, in both gypsy moths [23, 24, 26, 27] and other insects [28], and this variation is represented by the transmission term $\bar{\nu}\left[\frac{S(t)}{S(0)}\right]^{V}$, such that the initial mean transmission rate is $\bar{\nu}$ and the squared C.V. of transmission rates is V [29].

Using a Gillespie algorithm, it is straightforward to simulate a version of this SEIR model that allows for the effects of demographic stochasticity [25], the stochasticity due to small population sizes during the epizootic. Such a model, however, would not allow for the effects of drift due to population bottlenecks, or for stochastic population growth inside the host, nor would it allow for the possibility that hosts become infected by more than one virus strain. In allowing for demographic stochasticity, we therefore modified the stochastic version of the SEIR model such that the times between infection and death for individual hosts are generated through simulation of the within-host growth model, instead of being drawn from a gamma distribution. Also, the SEIR model assumes that all exposed hosts die, but in our stochastic

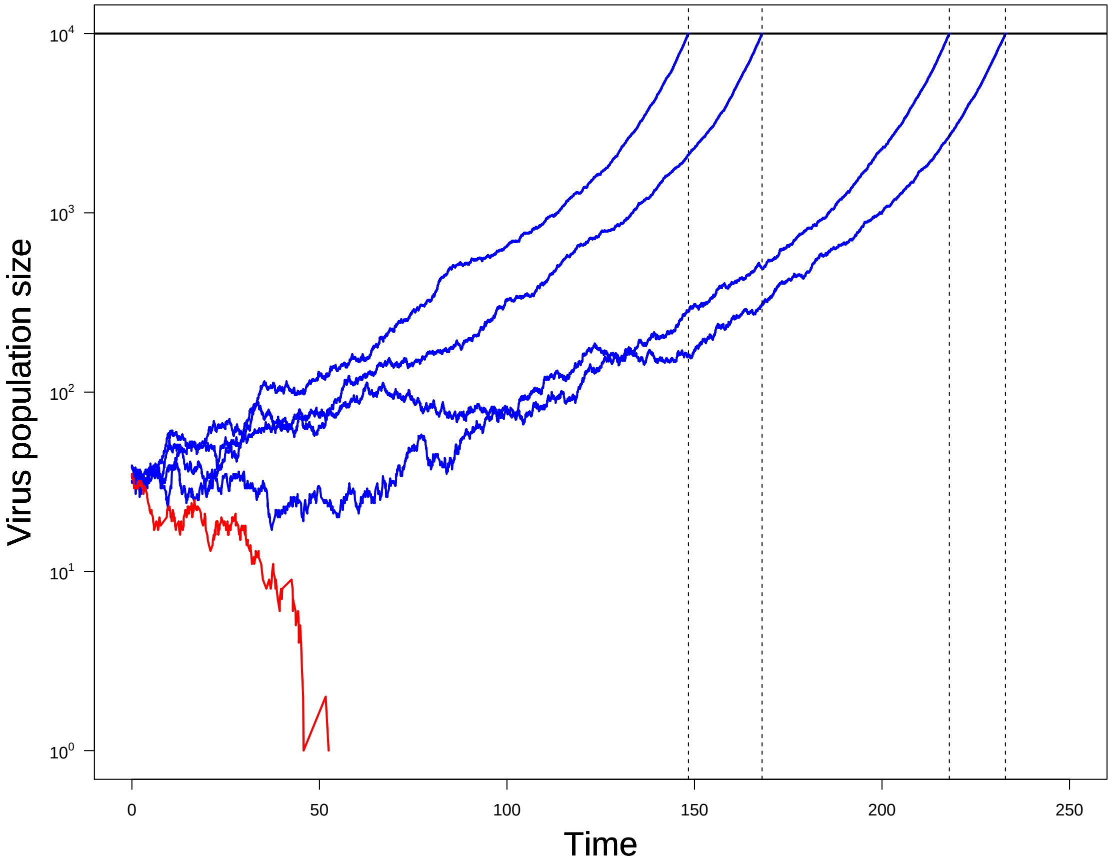

Figure S9: Realizations of the nonlinear birth death model (eqs. 5-8). In contrast to Fig S8, this model includes effect of the immune system. Parameter values are the same as in the nested-model simulations in the main text (table S3), except that to aid visualization, here we set the pathogen population size at host death $C = 10^4$ pathogen particles.

model, whether an exposed host dies or recovers is instead determined by the within-host growth model. The effect is that our within-host pathogen growth model is nested inside our version of the stochastic SEIR model. A final difference from the standard SEIR model is that we allow pathogen haplotypes to infect not just uninfected hosts, but also infected hosts, to allow for the possibility of multiple exposure events in the same host individual.

The Gillespie algorithm relies on the memorylessness of the exponential distribution, but in our nested model, the time until host death depends on the virus population within the host, leading to a distribution of times to death that is not memoryless. To simulate our model, we therefore instead use an algorithm developed by [30], which generalizes the Gillespie algorithm to allow for non-exponential event times. The steps in this algorithm are as follows: 1) Calculate the time at which the next exponentially distributed event will occur, as in the Gillespie algorithm; 2) Calculate the time at which the next non-exponentially distributed event will occur; 3) Use the minimum of 1 and 2 to determine the next event to occur, and update the time and the population sizes accordingly; 4) Return to step 1.

In the resulting model, there are three types of events; a host can contact the virus, a previously exposed host can die of an infection, or an infectious cadaver can cease to be infectious. The times to these respective events are;

$$
\begin{aligned}
t_e &\sim \text{Exponential}\left(\sum_{i=1}^{n_t} \bar{\nu} h_i I_t\right), && (13)\\
t_d &= \text{Min}(T_1, \ldots, T_l) - t, && (14)\\
t_r &\sim \text{Exponential}(\mu I_t). && (15)
\end{aligned}
$$

Here $T_1, \ldots, T_l$ are the times of death as predicted by the within-host model. Also, t_e is the time until the next host is exposed to the pathogen, t_d is the time until the next host dies of the pathogen, and t_r is the time until the next infectious cadaver decays and is therefore removed from the system.

Previous work has shown that constructing models that accurately describe baculovirus epizootics in gypsy moth populations requires an allowance for variation in infection risk across individuals, as in eqs. (9)-(12) [23, 29]. To adapt that model to allow for stochasticity, here we assume that $\bar{\nu} h_i$ is the infection risk of individual i, such that $\bar{\nu}$ is the average transmission rate, and h_i is a gamma-distributed random variate with mean 1 and coefficient of variation CV. The symbol I_t is the number of infectious cadavers at time t, n_t is the number of hosts that are alive at time t, l is the total number of exposed hosts in the population, and μ is the decay rate of the virus. Given these definitions, we calculate the time to the next event as the minimum of t_e, t_d and t_r.

Generating even a single realization of the full model requires a great deal of computing time. To increase computational efficiency, we therefore calculated times to death T_j and the accompanying within host virus dynamics by selecting within-host trajectories from a sample of 10^6 trajectories simulated before we simulated the full model. With this change, single realizations of the model can be completed within two days.

The nested epizootic model can be used to simulate the dynamics of the system within a single year, but to allow for long-term host-pathogen population dynamics, and thus pathogen evolution, we extended the model to allow for multiple host generations. Like many outbreaking forest insects [31], the gypsy moth has only one generation per year, and so our long-term model is based on a set of difference equations, which allow for natural selection and genetic drift in the host, over-wintering in the pathogen, and more generally for the long-term population dynamics of the host and the pathogen [24,29]:

$$N_{g+1} = 1 + \sum_{i=1}^{N_g} (\bar{\nu}_g h_i \lambda + b)\psi_i, \tag{16}$$

$$Z_{g+1} = 1 + \gamma Z_g + \phi \sum_{i=1}^{N_g} |\psi_i - 1|, \tag{17}$$

$$\bar{\nu}_{g+1} = \frac{1}{N_{g+1} - 1} \sum_{i=1}^{N_g} \bar{\nu}_g h_i (\bar{\nu}_g h_i \lambda + b)\psi_i. \tag{18}$$

Here, N_g and Z_g are the number of susceptible hosts and infectious cadavers in generation g, and $\bar{\nu}_g$ is the mean exposure risk of hosts in generation g. To explain the model, we begin with equations (16) and (17). On the right-hand side of these equations, we include a 1 to allow for the immigration of a single host and a single infectious cadaver in each host generation. Given that the number of uninfected hosts and infectious cadavers is generally above 10^3, this low level of migration had only very modest effects on the dynamics of the model, while nevertheless serving to prevent extinction of the host and the pathogen, which would otherwise have happened at least sporadically. In the host equation (16), the symbol combination $\bar{\nu}_g h_i$ is the realized exposure risk of host i in generation g, while b is the baseline reproductive rate of the host. Also, λ is the rate at which host fecundity increases with increasing host susceptibility, representing a tradeoff between fecundity and resistance that has been shown to occur in the gypsy moth [32,33]. The symbol ψ_i is an indicator variable, such that $\psi_i = 0$ indicates that host i has died from infection and $\psi_i = 1$ indicates that host i has survived to reproductive age, so that the term $\sum_{i=1}^{N_g} (\bar{\nu}_g h_i \lambda + b)\psi_i$ is the total reproductive output of the surviving hosts.

We use ψ_i again in equation (17), in which the summation $\sum_{i=1}^{N_g} |\psi_i - 1|$, is the total number of virus-killed hosts in generation g. The symbol ϕ describes the effective overwintering survival of cadavers produced in generation g, by which we mean that ϕ takes into account both the over-winter survival of infectious cadavers, and the high susceptibility of hatchling larvae in generation $g + 1$ [27], following previous work that showed that $\phi > 1$ [34]. The symbol γ is the overwintering survival of cadavers over longer time intervals.

Equation (18) describes the change in the mean exposure risk in the host population, which may be due either to selection for increased resistance during the epizootic, or to selection for increased fecundity during host reproduction (for simplicity, we assume that the host variation parameter CV is constant). Accordingly, the term $(\bar{\nu}_g h_i \lambda + b)\psi_i$ is the number of offspring

produced by host i. We then calculate the average exposure risk in the next generation by summing the expected risk for each of these offspring, $\bar{\nu}_g h_i$, and dividing by the total number of new offspring, $N_{g+1} - 1$, where the one accounts for the immigrant host.

It it is important to emphasize that our long-term model uses our stochastic SEIR model to calculate the number of hosts that survive the epizootic, and to calculate the number of hosts that are converted into infectious cadavers. The stochastic SEIR model is thus nested inside the long-term model, just as the within-host pathogen growth model is nested inside the SEIR model.

We simulated our models for 100 generations, because longer realizations produced nearly identical results. Because larvae can only be collected during outbreak years, when gypsy moth populations are at high densities, we extended each realization until the host population exceeded 10^4 and at least 2×10^3 hosts had died of the pathogen, to mimic the conditions under which our samples were collected. At the end of each realization, we recorded the composition of virus strains that comprised the pathogen population of each simulated virus-killed host.

To simulate within-host dynamics, we used parameter estimates from our previous work with a colleague [18], in which we fit the within-host model to speed of kill data from a dose-response experiment. Because the previous work was carried out in the lab, it did not provide an estimate of typical virus doses under field conditions. We therefore assumed an initial dose of $D = 10000$, which corresponds to $\approx 90\%$ of the maximum effective dose. This value of D is consistent with the observation that virus doses in the field tend to be very high [35], and our conclusions are nevertheless robust to this value (Supplemental Information D). All model parameters and their values are listed in table S3.

To simulate host-pathogen population dynamics, we used parameter estimates from previous work [24, 27]. The duration of each epizootic was 8 weeks, matching epizootics seen in gypsy moth populations in nature [36]. The value of the parameter λ determines the strength of selection for the increased fecundity that results from increased host infection risk, and it therefore affects the value of the average transmission rate $\bar{\nu}_g$ in generation g. Because the value of λ is poorly known, we selected $\lambda = 10^8$, which gave average fecundity values between 0.3 and 260 offspring per host, similar to the range in egg mass sizes typically observed in nature [37]. We set the host heterogeneity in susceptibility $CV = 2.5$, and the effective virus over-wintering parameter $\phi = 20$, each falling within the range of values calculated from previous work [24,27]. These values produced population cycles with a period of roughly 8 years and an amplitude of 3 orders of magnitude, matching the period and amplitude of cycles seen in nature [38,39].

In the purifying selection model, the probability of a host being susceptible to a particular virus strain was set to $\rho = 0.9$, based on previous dose-response data, which showed that the probability of a host being susceptible to a particular virus strain is at least $\rho = 0.97$, plus or minus 0.03 [40]. This estimate is close to $\rho = 1$, which would exactly replicate the model that lacks both transmission bottlenecks and replicative drift. To distinguish our selection model from this other model, we therefore assumed a value of ρ that was approximately 2 standard errors lower than its best empirical estimate. In Supplemental Information G, we show that

reducing the value of ρ improves the fit of the model to the data, but the selection model cannot compete with our best drift model for any reasonable value of ρ.

Note that all parameters were chosen before we compared the output of our models to our nucleotide-diversity data, and that the value of each parameter was the same in each of the four models that we compared to the nucleotide diversity data, with the exception of ρ, which was set to 1 in the neutral models and 0.9 in the purifying selection model. Adjusting the model likelihood values to allow for differences in the number of model parameters would therefore have no effect on model selection, since none of the model parameters are free parameters.

As we described in the main text, we considered four models. The most complex model includes both transmission bottlenecks and replicative drift, but we also constructed three alternative models by sequentially removing these two sources of genetic drift from the most complex model, and by adding purifying selection. First, we eliminated replicative drift by assuming that the ratio of pathogen strains at host death is the same as the ratio of pathogen strains that results from the transmission bottleneck. The resulting model thus assumes that, during the birth and death of virus particles within hosts, gene frequencies do not drift. This is often an implicit assumption in the literature, for example when changes in genetic diversity between transmission events are used to estimate infectious doses [41] or transmission bottleneck sizes [42, 43].

Second, we additionally eliminated the genetic drift caused by transmission bottlenecks, by assuming that the ratio of virus strains released at death is identical to the ratio of virus strains found in the cadaver that caused exposure. This latter model therefore assumes that the frequency of different virus strains are unchanged by either transmission bottlenecks or replicative drift, which is an implicit assumption whenever deterministic models are used to describe patterns of diversity [44]. Note that it is not possible to construct a model with replicative drift but without a transmission bottleneck, because replicative drift requires virus population sizes to be integer values, and forcing the virus population to have an integer value necessarily imposes a form of bottleneck.

Third, we added purifying selection into the model that lacked both transmission bottlenecks and replicative drift to determine whether non-neutral evolution is a better explanation for our data than drift. To implement purifying selection, as we described earlier, we added a single parameter ρ, which describes the probability that a host will be susceptible to a particular virus strain. Each host is therefore susceptible to a subset of x virus strains from the full set of 50 simulated virus strains, where x is binomial(50, ρ). If the cadaver that a host feeds on contains one or more strains to which the host is susceptible, then the host dies and the virus that it releases is a composite of the strains to which it was susceptible, with relative frequencies that match the relative frequencies in the cadaver. The model thus allows for selection within hosts, but neglects drift within hosts. Coinfections are implemented in the same way for all of our models.

Table S3: Model parameters, descriptions, values, units, and sources.

Parameter	Parameter description	Value	Units	Source
**Within host**				
β	Immune cell attack rate	4.70×10^{-6}	per virion per hour	[18]
r	Virus replication rate	0.21	per hour	[18]
c_1	Bottleneck parameter 1	42.7	virions	[18]
c_2	Bottleneck parameter 2	1228	virions	[18]
m	Initial immune-cell number	40738	lymphocytes	[18]
D	Applied dose	10000	virions	[35]
C	Threshold for death	1×10^9	virions	[21]
$\bar{x}_0$	Mean initial virus	$\frac{c_1 D}{c_2 + D} = 38.03$	virions	Calculated from above
**Within epizootics**				
V	Squared C.V. of transmission	6.25	dimensionless	[24]
μ	Cadaver decay rate	0.017	per hour	[27]
$\bar{\nu}$	Mean transmission rate	Varies across years	per larva per hour	[24]
ρ	Probability of host by strain susceptibility	0.9 or 1.0	per strain	[40]
**Between years**				
b	Baseline reproduction	0.2		[24]
λ	Fecundity resistance tradeoff	1×10^8	larvae hours	[37]
γ	Multi-year overwintering	0.2	dimensionless	[24]
ϕ	Singe-year overwintering	20	dimensionless	[27]

D Sensitivity of results to bottleneck size

In the main text, we assumed a mean bottleneck size of 38 virus particles (table S3) based on previous studies of baculovirus infections in the gypsy moth [18, 35]. When using this bottleneck size, the model that included transmission bottlenecks but not replicative drift was unable to explain our data, because virus populations within hosts tended to be too diverse. But tighter bottlenecks could reduce this diversity, and our estimate of bottleneck size of course has error associated with it. Here we show that the bottleneck-only model gives a relatively poor fit to the data even when bottleneck sizes are reduced by almost an order of magnitude.

The bottleneck size that we use to test the sensitivity of our model is derived from [42]. In this study, Zwart et al. infected *Spodoptera exigua* larvae with tagged strains of *Autographica california* multiple nucleopolyhedrovirus. Using a statistical model that related loss of virus diversity to bottleneck size, Zwart et al. found that the mean bottleneck size was approximately 4.8 virus particles.

To test the bottleneck-only model with this narrower bottleneck estimate, we made two adjustments to our original bottleneck-only model. First, we altered the dose of virus that hosts consume from 10,000 occlusion bodies to 156 occlusion bodies (see table S3). This change reduced the average bottleneck size from 38 to 4.8, thereby implementing the Zwart et al. estimate of the bottleneck. This reduction in dose, however, also caused mortality rates given exposure to drop from 98% to 34%. This reduction is highly unrealistic given that, in the Zwart et al. data, the mortality rate given exposure was near 100%, as mortality rates typically are for baculovirus experiments that use field-relevant virus doses. We therefore also altered the model to maintain our original rate of mortality given exposure ($\approx 98\%$). To do this, we identified simulated pathogen trajectories that resulted in host survival, and we replaced them with new simulated trajectories in which death occurred, as necessary to maintain a recovery rate of no more than 2%.

In Fig S10, we show that the distribution of within host diversity for this model can indeed explain the data better than our original bottleneck-only model (compare to Fig 3). Even using this tighter-bottleneck, however, the bottleneck-only model does not explain the data as well as the full model, which also includes replicative drift, because the tighter-bottleneck model predicts that more samples will have high nucleotide diversity (≈ 0.2) and fewer samples will have intermediate levels of nucleotide diversity ($\approx 0.04 - 0.09$) than compared to the data. This lack of fit is reflected in the likelihood estimates (median log likelihood estimates: Original bottleneck (38) $= -266.7$, Zwart et al. bottleneck (4.8) $= -72.7$, Full model $= -63.9$). The likelihood estimates for the Zwart-et-al.-bottleneck-only model over 100 Monte Carlo simulations ranged from -75.8 to -70.1, and as table S4 shows, this range is not large enough to cast any doubt on our conclusion that the full model better explains the data.

Note that there is a large discrepancy between the Zwart et al. estimate of the bottleneck

size and the Kennedy et al. estimate of the bottleneck size, a discrepancy that is larger than the uncertainty in the estimate from either study. There are two possible explanations for such a large discrepancy. The first is the obvious difference that the studies used different virus species and host species, the importance of which is unknown. The second is that the Zwart et al. estimate is derived from data on loss of virus diversity using a statistical model that assumes that all lost diversity can be attributed to a transmission bottleneck. Our models demonstrate that diversity may also be lost due to replicative drift, and so the Zwart et al. estimate may be overestimating the severity of transmission bottlenecks. The Kennedy et al. estimate is instead derived from fitting models to data on mortality and time of death [18], and so the estimate is not confounded by replicative drift. For these reasons, we used the estimate of Kennedy et al. in the main text. Although unrealistically severe bottlenecks provide an explanation for our data that is at least not terrible, we argue that a better explanation for the data is that both replicative drift and transmission bottlenecks shape the diversity of the gypsy moth baculovirus.

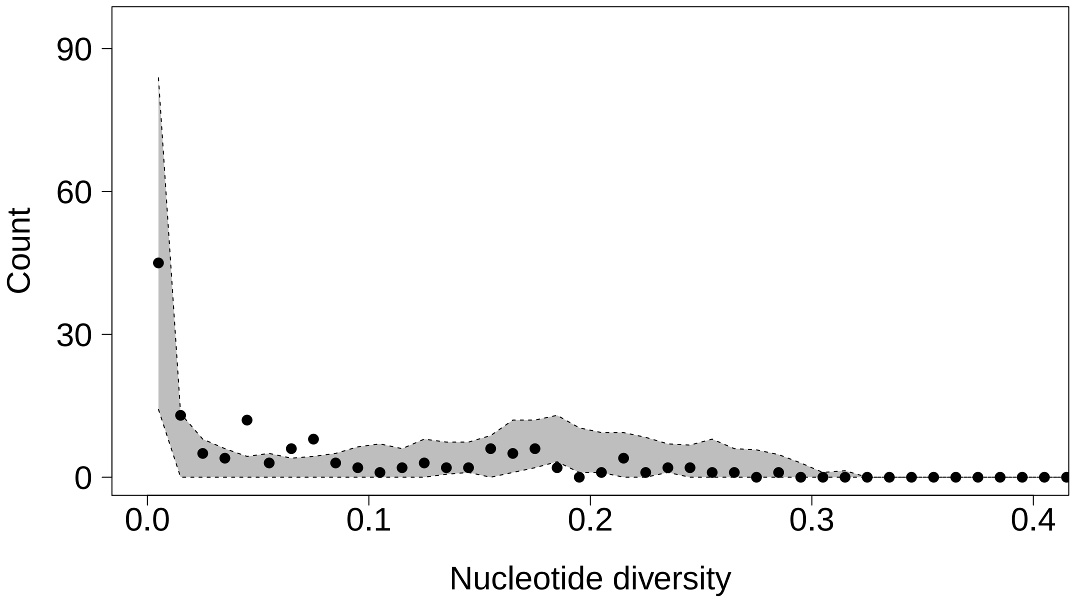

Figure S10: Nucleotide diversity predicted by a bottleneck only model that uses the estimate of effective bottleneck size from [42]. This model provides a better explanation for the data than the original bottleneck-only model, but a worse fit to the data than the full model that includes both bottlenecks and replicative drift. We attribute the improvement in performance to the fact that the bottleneck estimate generated by Zwart and colleagues is in reality an estimate of effective bottleneck size, rather than the number of virus particles that initiate infection. It therefore implicitly allows for effects of both transmission bottlenecks and replicative drift, without explicitly describing either. Values underlying data points are provided in S1 Data.

E Model of *de novo* mutation

A possible alternative explanation for our data is that diversity within hosts arose through the accumulation of mutations during pathogen replication within hosts. This seems unlikely given both that double stranded DNA viruses have low mutation rates and that variation is concentrated at a small number of loci, but to be thorough, we consider whether a model of *de novo* mutation can explain our data.

The mutation model follows a branching process with finite alleles and finite sites. We assume that each infection begins as a single virus particle. Each virus particle then doubles each virus generation, until the total virus population size reaches size C, and host death occurs. During each doubling, new mutations occur at rate M per locus. To be consistent with our data, we assume that there are 712 loci. Note that this is equivalent to having a larger genome in which strong purifying selection only allows variants to persist at a pre-specified 712 sites. Mutations are assumed to be selectively neutral, but only two alleles exist for any locus. If a second mutation occurs at a site that has already mutated, this mutation is a reversion back to the original allele. If the total number of mutations at a locus is an odd number, the virus thus has a mutant allele, and if the total number of mutations is an even number, the virus has the founder allele. This assumption has no qualitative effect on our results.

For each model simulation, we calculated the mean nucleotide diversity of the virus population within each host. These values were then compared to the mean nucleotide diversity in the sequence data, as with our other models. This model does not include replicative drift, concurrent transmission of multiple strains, or reinfection, and therefore it attempts to explain within host diversity through mutation alone. A lack of fit to the data would therefore suggest that one or more of these missing mechanisms are necessary to explain pathogen diversity in our data.

Due to computational constraints on memory and time, we were only able to simulate 15 rounds of virus replication. We assumed that host death occurs after round 15, which corresponds to a virus population size at host death $C = 32768$ (half of the 30 rounds of doubling necessary for 1 founder to achieve a population size exceeding 10^9). To confirm that our conclusions were not influenced by the number of rounds of replication, we additionally simulated the model assuming death after 5 and 10 rounds, but these changes had no qualitative impact on our conclusions. We simulated the model using mutation rate $M = 10^{-7}$, which is the best estimate of the per nucleotide mutation rate for double-stranded DNA viruses [22]. Because we saw very little virus diversity within any hosts when using this parameter value, we reran our simulations using mutation rates of 10^{-1}, 10^{-2}, and 10^{-3}. These higher mutation rates generated substantial diversity within hosts, but all hosts were infected with highly diverse virus populations (Fig S11). As a result, none of these parameter sets were able to reproduce the variation in diversity in the data, with some hosts being infected by virus populations of high

diversity and other hosts infected by virus populations with low diversity. In this model, all hosts had similar levels of nucleotide diversity. The model is therefore unable to explain our data, and so we conclude that *de novo* mutation is an unlikely explanation for the data.

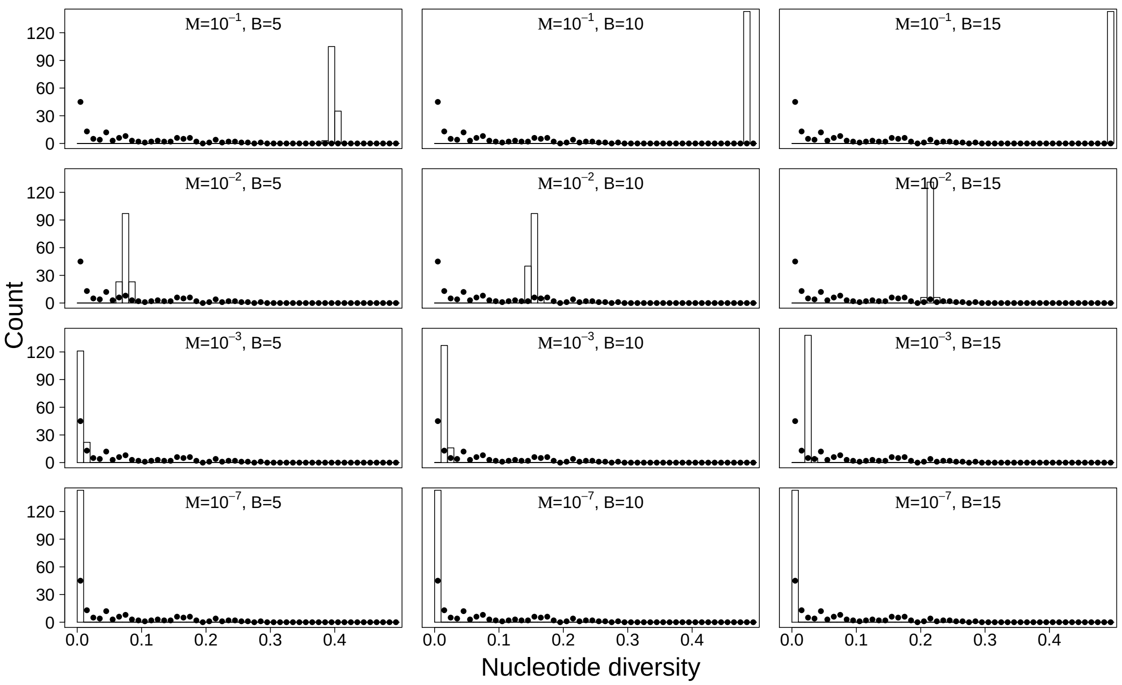

Figure S11: The distribution of nucleotide diversity. Points show the data, and bars show the predictions generated by a model of *de novo* mutation. Each row uses a different mutation rate M, and each column uses a different number of replication rounds B. Note that very little diversity is generated at realistic mutation rates (i.e. bottom row). Even when mutation rates are high, the model is unable to explain the distribution of diversity seen in the data, because the model predicts that hosts will have very little variation in their nucleotide diversity relative to that seen in our data. Values underlying data points are provided in S1 Data.

F Likelihood Calculations

To calculate a likelihood score for each model, we compared the prediction of nucleotide diversity within hosts from each model to the distribution of nucleotide diversity in the data. Each realization of each model produces a matrix representing the frequency of each of 50 pathogen strains in each of 2000 randomly-selected hosts killed by the pathogen in the final year of the model realization. The cells of this matrix thus store the fraction of a simulated cadaver that is made up of a particular virus strain.

To compare the model predictions to the sequence data, we first assigned genotypes to each of the virus strains. In our sequence data there were 712 sites with allele frequencies between 0.05 and 0.95, and so we generated 712 segregating sites from the model output. To do this, we constructed pathogen strains one segregating site at a time, by selecting a uniform random variate between 0.05 and 0.95, and assigning genotype "0" to that fraction of the pathogen strains, and genotype "1" to all other strains. Strains were thus assigned randomly to each group. We repeated this process until we had constructed genotypes composed of 712 segregating sites.

A common approach when working with this type of data is to calculate a composite likelihood, sometimes referred to as a psuedo-likelihood [45]. A composite likelihood is the likelihood that results when partial likelihoods are calculated for subsets of a large dataset, and then the components of the likelihood are combined. In the population genetics literature, composite likelihoods often arise when partial likelihoods are calculated at each locus independently and then combined into a single value (for example, [46]). This calculation would match the true likelihood if all loci were unlinked and thus independent, but if loci are linked and thus not independent, there can be a large difference between the composite likelihood and the true likelihood, potentially leading to incorrect inferences. We avoided this problem by calculating likelihood estimates based on a summary statistic, the nucleotide diversity, instead of calculating likelihoods at individual loci (in Supplemental Information I, we show that our conclusions hold for other summary statistics). Our summary statistic covers the full set of data that we analyzed, and so our approach avoids the problem with pseudo-replication that can arise when the assumption of independence between loci is violated. By using a summary statistic to describe complex data, we may have sacrificed statistical power, but the differences in log likelihood between our models were on the order of 200 log units, and so statistical power was not an issue.

To calculate likelihoods, we randomly selected 1000 infected hosts from our model output, and we calculated the nucleotide diversity of each simulated host using the simulated virus haplotypes described above. We used these simulated data to estimate the likelihood of observing the actual data for any particular model realization. In practice, we first recorded the number of cadavers whose nucleotide diversity fell in each of 50 bins ranging in value from 0 to 0.5 by increments of 0.01. Doubling or halving the bin widths had no impact on our conclusions. We used these numbers to estimate the probability that the nucleotide diversity of any particular cadaver from our sequence data would fall in any particular bin. To avoid probability values of 0 that result from using a finite number of simulated hosts, we slightly adjusted the probability of

each bin by adding 1 to each bin before dividing by the number of simulated hosts (1000) plus the number of bins (50). This is a conservative approach in that it slightly improves the likelihood of poorly fitting models relative to better fitting models. We used these probability values in a multinomial distribution using "dmultinom" in R, to generate a Monte Carlo estimate of the likelihood for each realization of each model.

Following [47], we averaged likelihood estimates across realizations. The computational constraints of simulating the models were quite severe, but likelihood differences between models were large enough that 69 realizations (68 realizations for the purifying selection model) were sufficient to establish that the best model is vastly better than the alternative models. Although our likelihood estimates depend on the random assignment of genotypes and simulated infected hosts, in practice the variation across realizations of the genotype assignment process was very small compared to the difference in mean likelihoods between models, and so this variation had no effect on our results. To show this, we repeated the genotype assignment process 100 times, re-calculating the likelihood each time. As table S4 illustrates, the ordering of the models would be the same even if we had only the worst likelihood for the best model, and the best likelihoods for the other models.

Table S4: Minimum, median, and maximum log-likelihood estimates for each of our models, across 100 realizations of the genotype-assignment process used in our likelihood calculations.

**Model**	**Minimum**	**Median**	**Maximum**
Purifying selection	-364.6	-353.1	-338.3
Bottlenecks only	-274.9	-266.7	-248.5
Bottlenecks and replicative drift	-66.0	-63.9	-61.6
Neither bottlenecks nor drift	-508.3	-503.2	-498.5

G Effects of varying the strength of selection in the purifying selection model

In our selection model, the probability of a particular host being susceptible to a particular virus strain is equal to the parameter ρ. This value is based on a previous bioassay experiment [40], in which $97\% + / - 3\%$ of hosts died from exposure to a plaque purified virus strain when fed a realistic dose on a leaf disk, a typical result in this system [26]. Because setting $\rho = 1.0$ recreates a neutral model, and because the impact of selection increases as ρ is reduced, we used $\rho = 0.9$ in our simulations of the selection model. This value is two standard errors less than its best estimate [40].

In Fig 3 and table S4, we show that a purifying selection model using this parameter value provides an extremely poor fit to the data. Here we test whether other values of ρ can explain our data. We reran our selection model using $\rho = 0.8$, $\rho = 0.7$, and $\rho = 0.6$. The fit improves as we lower ρ, but to achieve a likelihood score and model fit that is almost as good as our best model requires that we use the very unrealistic value $\rho = 0.6$, which is about 12 standard errors smaller than the best estimate of this parameter (Fig S12 and table S5).

To illustrate how unrealistic this parameter value is, we consider our model in a Bayesian framework, in which the posterior probability of ρ is discounted by its prior probability. If the prior for ρ is based on its empirical estimate from [40], then at $\rho = 0.6$, the prior density is approximately 70 log units smaller than at $\rho = 0.90$, and 72 log units smaller than at $\rho = 1.0$. The effective value of ρ is 1.0 in our drift-only models, because all larvae are susceptible to all virus strains. The likelihood of the selection model at $\rho = 0.6$ should therefore be discounted by approximately 72 log units when compared to the likelihood of our neutral models. We therefore conclude that neutral evolution is a better explanation for our data than selection.

Table S5: Minimum, median, and maximum log-likelihood estimates for selection models using smaller values for host by virus strain susceptibility ρ. As in table S4, these likelihood estimates were generated using 100 realizations of the genotype-assignment processes.

Model	Minimum	Median	Maximum
$\rho = 0.8$	-219.7	-209.8	-201.1
$\rho = 0.7$	-104.8	-100.8	-92.0
$\rho = 0.6$	-68.4	-66.0	-63.1

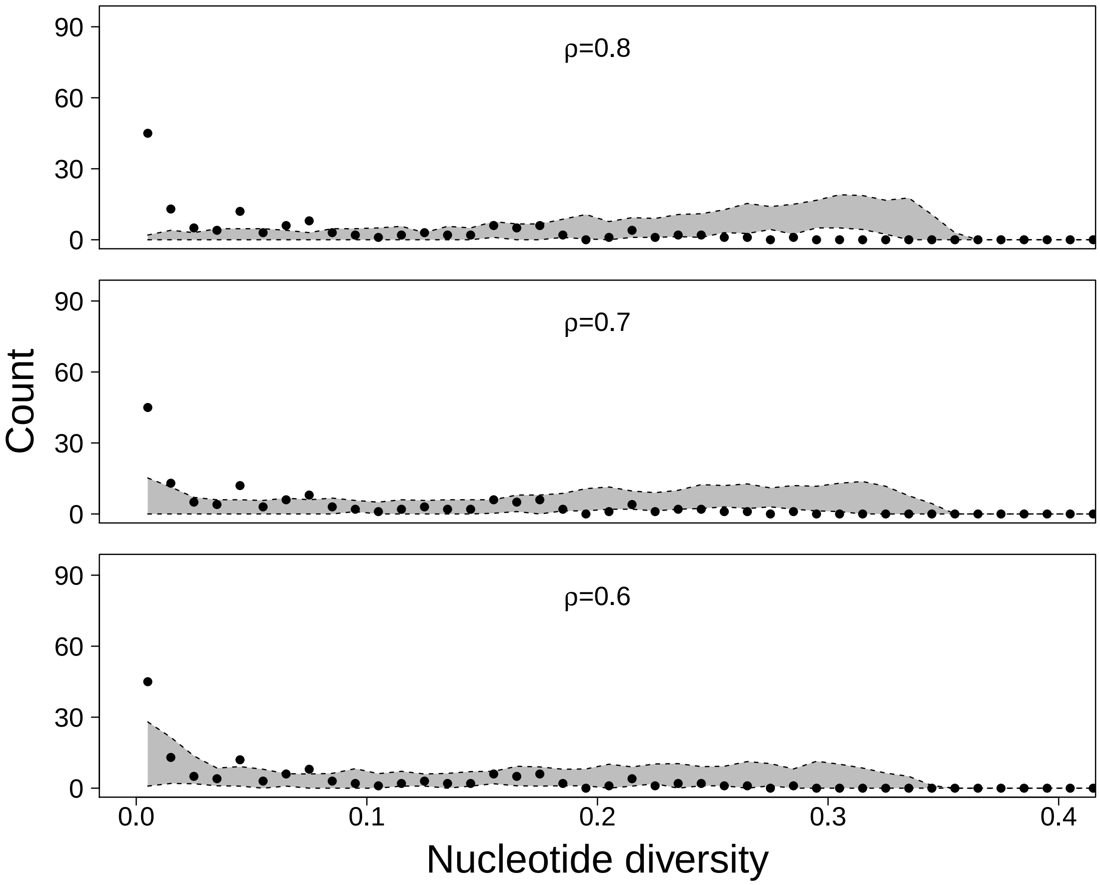

Figure S12: Plots of nucleotide diversity. As in Fig 3, the grey interval shows 95% confidence intervals of model simulations and black points show the results from our sequence data. The three figures from top to bottom present the results from models using different estimates of host by virus strain susceptibility $\rho = 0.8$, $\rho = 0.7$, and $\rho = 0.6$. The current best empirical estimate is $\rho = 0.97$. In the main text, we showed that when $\rho = 0.9$, the model predicted levels of diversity within hosts that were too high, and here we show the sensitivity of our conclusion to the value of ρ. The selection model consistently predicts levels of nucleotide diversity within hosts that are too high even for values of ρ that are unrealistically small (i.e. up to 12 standard errors from its best estimate). Values underlying data points are provided in S1 Data.

H Testing for evidence of selection within hosts

As we have shown, our simple purifying selection model is a poor explanation for the data, but we have not ruled out that more complex models of selection might perform better. Here, we use a method similar to the McDonald-Kreitman test [48] to determine whether there is evidence that selection shapes pathogen diversity within hosts.

Like the McDonald-Kreitman test, our method compares the relative representation of synonymous and non-synonymous variation at loci that are segregating or not segregating within a population (in our case, "population" refers to the virus population within a host). Assuming that selection disproportionately affects loci with non-synonymous variants, directional selection should cause non-synonymous alleles to fix within a population faster than synonymous alleles. Frequency dependent selection, which might result for example from immune-mediated diversifying selection, should alternatively prevent non-synonymous alleles from fixing in a population. We can therefore test whether selection is acting within hosts by examining whether loci with non-synonymous variants segregate at higher or lower rates within hosts relative to loci with synonymous variants than would be expected by chance. Note, however, that linkage may limit the ability of selection to act independently on synonymous and non-synonymous mutations, and so this test is imperfect. Strong signals of selection may nevertheless emerge if they are present.

To implement this test, we needed to identify which of our 712 segregating sites could be classified as "synonymous" or "non-synonymous". First, we removed all of the sites with indels or with more than two alleles. We then constructed a custom database for "snpEff" [49] using the gypsy moth baculovirus whole genome sequence on GenBank [6], and we ran "snpEff" to determine which of the remaining sites contained synonymous or nonsynonymous coding variants. Variants in non-coding parts of the genome were ignored, leaving us with 289 synonymous sites and 251 non-synonymous sites.

If selection were not acting on the variants within hosts, we would expect the ratio of synonymous to non-synonymous sites within hosts to have a ratio that is not statistically different from 289:251 both for sites segregating within a host and for sites fixed within a host. We defined segregating sites as sites where both alleles occurred at frequency greater than 0.025, and non-segregating sites as sites where this was not true. We then tested for selection by performing a series of Fisher exact tests implemented using the function "fisher.test" in "R" [50]. One test was performed for each sample resulting in 143 test statistics.

After Bonferroni correction [51], none of the 143 tests were statistically significant. Without correcting for multiple testing, 9 of 143 tests yielded p-values less than 0.05, which is close to the null expectation of 7.15. We therefore conclude that there is no evidence of selection acting within hosts.

I Alternative summary statistics

In the main text, we compared model predictions to sequence data using mean nucleotide diversity π. Here we show that using the effective number of alleles A_e, the proportion of polymorphic loci P, or a metric that we refer to as relative nucleotide diversity $\hat{\pi}$ yields the same conclusions as we found using nucleotide diversity.

The effective number of alleles A_e is the number of alleles that would be required to explain a given level of genetic diversity, assuming that the alleles occur at equal frequency. In a population with very low genetic diversity, the effective number of alleles would be close to one, and it would increase as the genetic diversity in the population increases. We calculated the mean effective number of alleles in each of our samples using the following formula.

$$A_e = \frac{1}{n} \sum_{j=1}^{n} \left(\frac{1}{\sum_{i=1}^{k_j} x_{ij}^2} \right). \tag{19}$$

Here n is the number of loci, k_j is the number of alleles at locus j, and x_{ij} is the frequency of allele i at locus j.

We also used the proportion of polymorphic sites P, which is the fraction of sites that are segregating within a population. Here we consider a site to be segregating within a sample if the frequency of the major allele was less than 0.99.

Lastly, to quantify the diversity present at the within-host scale relative to the diversity present at the between-host scale, we use a summary statistic $\hat{\pi}$, or relative nucleotide diversity. This statistic describes the level of nucleotide diversity present within hosts relative to the level of nucleotide diversity present across consensus sequences across hosts, and it is calculated by dividing observed nucleotide diversity within hosts by observed nucleotide diversity across consensus sequences. Note that this value cannot be negative, but is otherwise unbounded, because high levels of coinfection can generate high nucleotide diversity within hosts but low levels of nucleotide diversity between consensus genome sequences.

For all three summary statistics, we restricted our analysis to the 712 sites previously identified as segregating at the population level. We analyzed these data identically to the nucleotide diversity data, with two modifications. First, the plausible range of these summary statistics differed from nucleotide diversity, and so in our calculation of the likelihood, we altered the bins so that instead of ranging from 0 to 0.5, they ranged from 1 to 1.7 for the effective number of alleles A_e, from 0 to 1 for the proportion of polymorphic sites P, and from 0 to 15 for relative nucleotide diversity $\hat{\pi}$. The width of each bin was modified to maintain 50 total bins. Second, to save computational time, the likelihood was estimated over 10 realizations of the genotype-assignment process instead of the 100 realizations that we used for nucleotide diversity. The differences in likelihood estimates between models are nevertheless clear.

The likelihood estimates that arise when using different summary statistics are presented in separate tables, because the different summary statistics arise from different data and therefore cannot be directly compared to each other. Nevertheless, these three new tables show the

same qualitative results as table S4; the model that includes both transmission bottlenecks and replicative drift is our best model (tables S6-S8 and figs. S13-S15).

Table S6: Minimum, median, and maximum log-likelihood estimates for models when the test statistic is the effective alleles A_e.

**Model (Summary statistic A_e)**	**Minimum**	**Median**	**Maximum**
Neither bottlenecks nor drift	-502.4	-498.5	-495.4
Bottlenecks only	-271.3	-265.0	-260.8
Bottlenecks and replicative drift	-81.2	-79.6	-77.3
Purifying selection	-361.6	-351.8	-347.9

Table S7: Minimum, median, and maximum log-likelihood estimates for models when the test statistic is the proportion of polymorphic sites P.

**Model (Summary statistic P)**	**Minimum**	**Median**	**Maximum**
Neither bottlenecks nor drift	-522.9	-514.1	-511.1
Bottlenecks only	-318.7	-305.8	-298.8
Bottlenecks and replicative drift	-121.1	-117.0	-113.4
Purifying selection	-447.1	-416.9	-412.1

Table S8: Minimum, median, and maximum log-likelihood estimates for models when the test statistic is the relative nucleotide diversity $\hat{\pi}$.

**Model (Summary statistic $\hat{\pi}$)**	**Minimum**	**Median**	**Maximum**
Neither bottlenecks nor drift	-592.4	-591.5	-589.6
Bottlenecks only	-316.0	-312.5	-309.1
Bottlenecks and replicative drift	-13.3	-13.2	-13.1
Purifying selection	-347.7	-346.8	-344.8

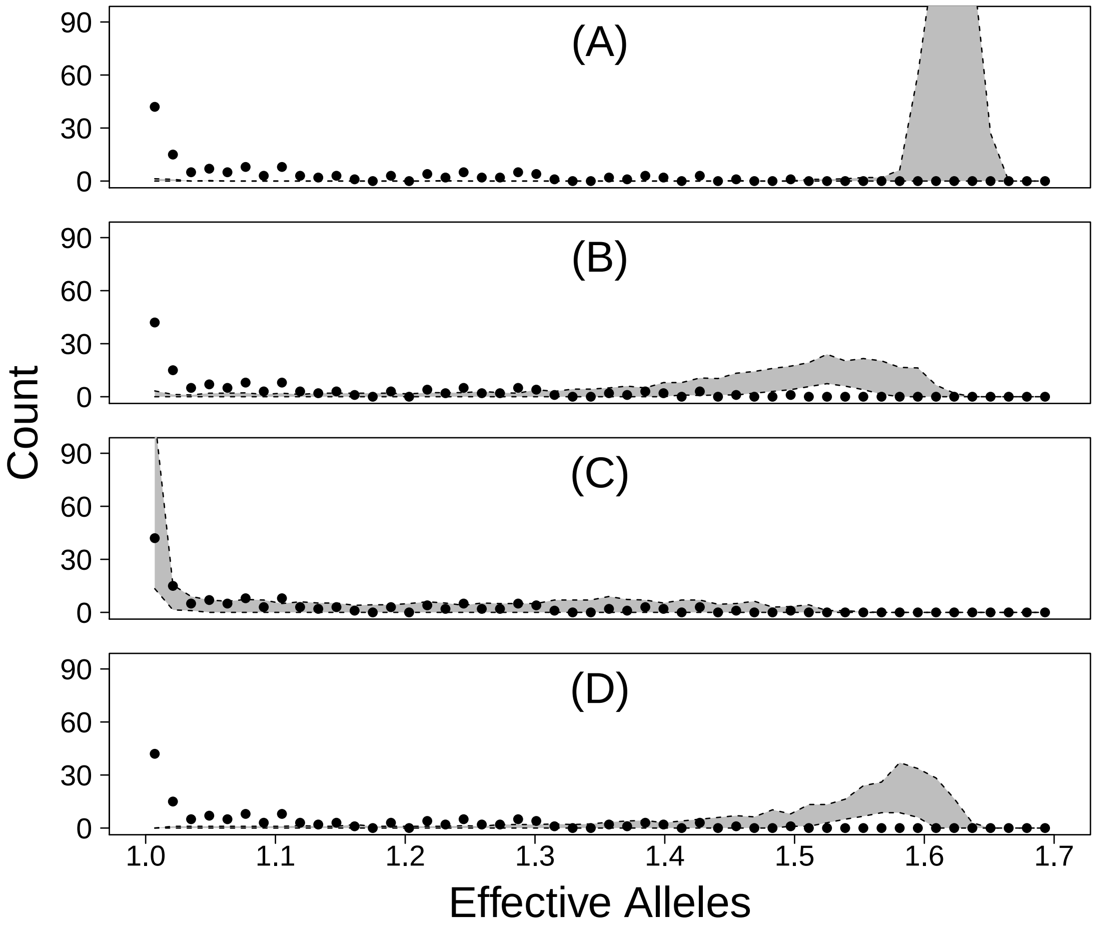

Figure S13: Fit of model predictions to data when using effective alleles A_e instead of nucleotide diversity π. As in Fig 3, grey shading shows 95% prediction envelopes of the model and points represent measures from the sequence data. (A) shows the model that includes neither transmission bottlenecks nor replicative drift, (B) shows the model that includes transmission bottlenecks but lacks replicative drift, (C) shows the model that includes both transmission bottlenecks and replicative drift, and (D) shows the model that includes purifying selection but lacks transmission bottlenecks and replicative drift. The model in panel (C) clearly fits the data best. Values underlying data points are provided in S9 Data.

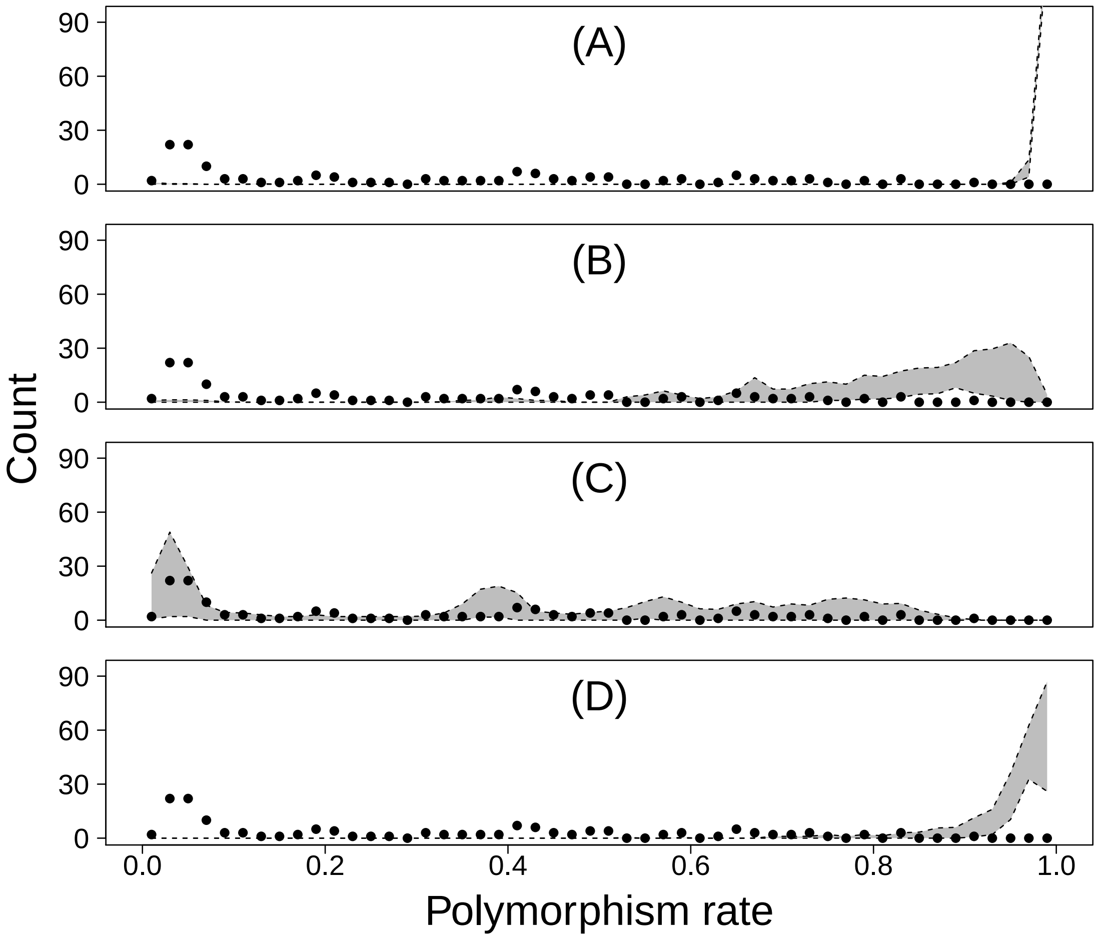

Figure S14: Fit of model predictions to data when using the proportion of polymorphic sites P, formatted as in Fig S13. The model that includes both transmission bottlenecks and replicative drift (panel C) again provides by far the best fit to the data. Values underlying data points are provided in S10 Data.

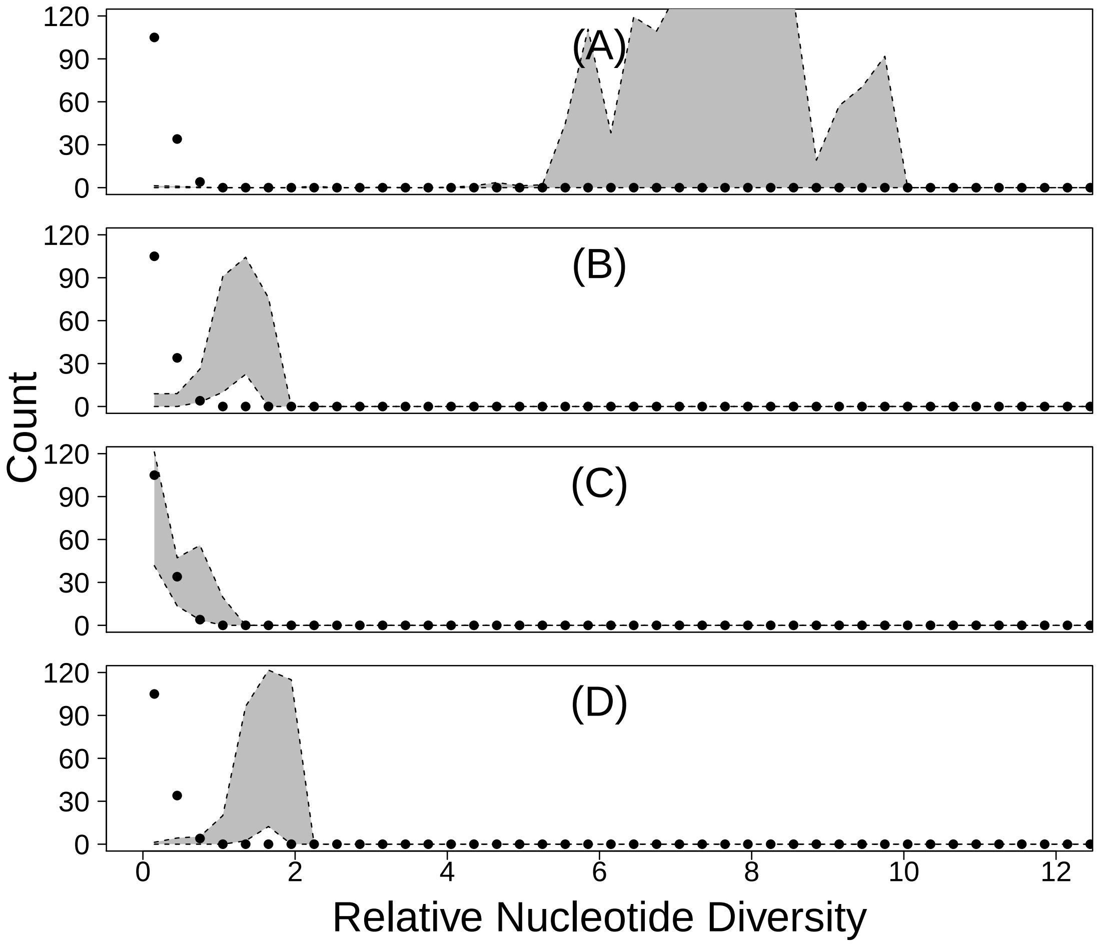

Figure S15: Fit of model predictions to data when using the relative nucleotide diversity $\hat{\pi}$, formatted as in Fig S13. Panel C again provides the best fit to the data. Values underlying data points are provided in S11 Data.

J Mapping error

In Fig 4 in the main text, the distributions of allele frequencies in the data differ from those predicted by the best model in two ways. First, the tails in the data histograms tend to be longer than the tails in the model histograms. Second, the peaks around intermediate frequency alleles in the data histograms are much broader than the peaks in the model histograms. Here we show that both of these features can be explained by biases introduced during the mapping of short reads to a reference genome.

We begin by showing the effect of mapping biases on the distribution of allele frequencies in a host infected by a single pathogen genotype. This required a three-step process. First, we created a synthetic pathogen genotype, by combining the reference genome [6] with the allelic variants that we detected in our sequence data. In practice, we used a probability of 0.5 that any particular allele would match the alternative sequence rather than the reference. Second, using this synthetic genotype, we simulated Illumina sequencing by generating 5×10^5 short sequence reads, each 100 bp long, from the generated pathogen genotype, while including a 2% sequencing error rate. Third, we mapped the synthetic Illumina-like sequence reads back to the reference genome in the same way that we mapped the real sequence data. The results show that, if sequencing error is sufficiently high, then a frequency histogram with a peak at allele frequencies of less than 1 can be generated even when the host is infected by only a single pathogen strain (Fig S16).

Next, we showed that the spread around peaks at intermediate frequencies in histograms of allele frequencies is partly due to mapping errors. To do this, we used a very similar protocol to the one we just described, except that in this case, rather than generating a single pathogen strain, we generated three synthetic strains. For these three synthetic strains, we assumed a probability of 0.1 that any particular allele would match the alternative sequence rather than the reference. The host was then assumed to be co-infected by these three pathogens at a ratio of 4:3:3. The results show that the errors introduced during mapping lead to erroneously broad peaks, instead of the tight clusters of intermediate frequency alleles that we would expect in the absence of errors. Allowing for mapping error in this way yields histograms of allele frequencies that closely match the data histograms (compare Fig S17 to Fig 4 in the main text).

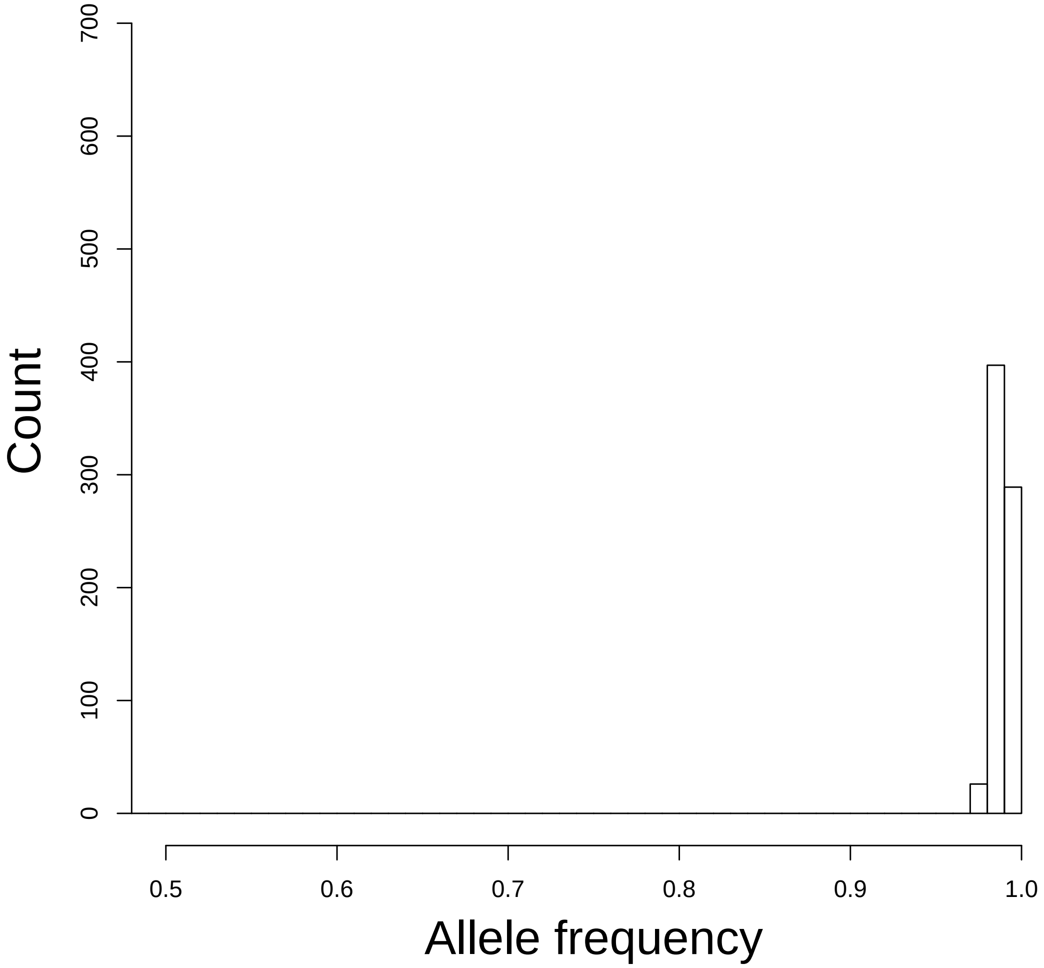

Figure S16: Histogram of synthetic sequence data from a single-infected host, as produced by our model with the inclusion of mapping errors. Values underlying histogram are provided in S12 Data.

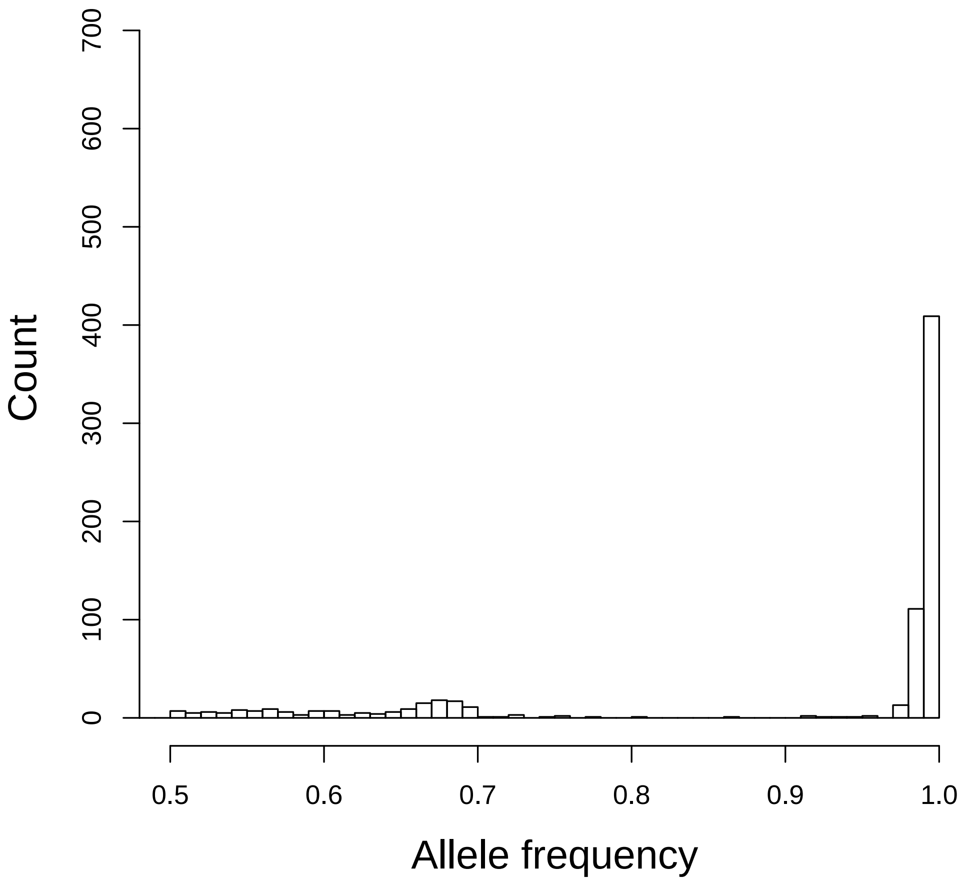

Figure S17: Histogram of synthetic sequence data from a co-infected host, as produced by our model with the inclusion of mapping error. Values underlying histogram are provided in S13 Data.

References and Notes

[1] Bell RA, Owens CD, Shapiro M, Tardif JR. Mass rearing and virus production. In: Doane CC, McManus ML, editors. The Gypsy Moth: Integrated Pest Management. Washington, DC: USDA Technical Bulletin; 1981. p. 599–655.

[2] Rohrmann GF. Baculovirus Molecular Biology. Bethesda: National Library of Medicine (US); 2008.

[3] Christian PD, Gibb N, Kasprzak AB, Richards A. A rapid method for the identification and differentiation of *Helicoverpa* nucleopolyhedroviruses (NPV *Baculoviridae*) isolated from the environment. J Virol Methods. 2001;96(1):51–65.

[4] Sambrook J, Fritsch EF, Maniatis T. Molecular Cloning: A Laboratory Manual. New York: Cold Spring Harbor Laboratory Press; 1989.

[5] Meyer M, Kircher M. Illumina sequencing library preparation for highly multiplexed target capture and sequencing. Cold Spring Harb Protoc. 2010;2010(6):1–10.

[6] Kuzio J, Pearson MN, Harwood SH, Funk CJ, Evans JT, Slavicek JM, et al. Sequence and analysis of the genome of a baculovirus pathogenic for *Lymantria dispar*. Virology. 1999;253:17–34.

[7] Langmead B, Salzberg SL. Fast gapped-read alignment with Bowtie 2. Nat Methods. 2012;9(4):357–359.

[8] Li H, Handsaker B, Wysoker A, Fennell T, Ruan J, Homer N, et al. The sequence alignment/map format and SAMtools. Bioinformatics. 2009;25(16):2078–2079.

[9] Li H. A statistical framework for SNP calling, mutation discovery, association mapping and population genetical parameter estimation from sequencing data. Bioinformatics. 2011;27(21):2987–2993.

[10] Koboldt DC, Zhang Q, Larson DE, Shen D, McLellan MD, Lin L, et al. VarScan 2: somatic mutation and copy number alteration discovery in cancer by exome sequencing. Genome Res. 2012;22(3):568–576.

[11] Langmead B, Trapnell C, Pop M, Salzberg SL. Ultrafast and memory-efficient alignment of short DNA sequences to the human genome. Genome Biol. 2009;10(3):R25.

[12] Fujita PA. Combining models with empirical data to examine dispersal mechanisms in the gypsy moth nucleopolyhedrosis host-pathogen system [Ph.D. Dissertation]. University of Chicago; 2007.

[13] Nei M, Li WH. Mathematical model for studying genetic variation in terms of restriction endonucleases. Proc Natl Acad Sci USA. 1979;76(10):5269–5273.

[14] Kot M. Elements of Mathematical Ecology. Cambridge: Cambridge University Press; 2001.

[15] Renshaw E. Modeling Biological Populations in Space and Time. Cambridge: Cambridge University Press; 1991.

[16] Saaty TL. Some stochastic-processes with absorbing barriers. J R Stat Soc Series B Stat Methodol. 1961;23:319–334.

[17] Kennedy DA, Dukic V, Dwyer G. Pathogen growth in insect hosts: inferring the importance of different mechanisms using stochastic models and response-time data. Am Nat. 2014;184(3):407–423.

[18] Kennedy DA, Dukic V, Dwyer G. Combining principal component analysis with parameter line-searches to improve the efficacy of Metropolis–Hastings MCMC. Environ Ecol Stat. 2015;22(2):247–274.

[19] Ashida, Brey PT. In: Brey PT, Hultmark D, editors. Molecular Mechanisms of Immune Responses in Insects. London: Chapman & Hall; 1998.

[20] Trudeau D, Washburn JO, Volkman LE. Central role of hemocytes in *Autographa californica M* nucleopolyhedrovirus pathogenesis in *Heliothis virescens* and *Helicoverpa zea*. J Virol. 2001;75(2):996–1003.

[21] Shapiro M, Robertson JL, Bell RA. Quantitative and qualitative differences in gypsy moth (Lepidoptera: Lymantriidae) nucleopolyhedrosis virus produced in different-aged larvae. Journal of Economic Entomology. 1986;79:1174–1177.

[22] Sanjuán R, Domingo-Calap P. Mechanisms of viral mutation. Cell Mol Life Sci. 2016;73(23):4433–4448.

[23] Dwyer G, Elkinton JS, Buonaccorsi JP. Host heterogeneity in susceptibility and disease dynamics: Tests of a mathematical model. Am Nat. 1997;150(6):685–707.

[24] Elderd BD, Dushoff J, Dwyer G. Host-Pathogen Interactions, Insect Outbreaks, and Natural Selection for Disease Resistance. Am Nat. 2008;172:829–842.

[25] Keeling MJ, Rohani P. Modeling Infectious Diseases. New Jersey: Princeton University Press; 2008.

[26] Dwyer G, Firestone J, Stevens TE. Should models of disease dynamics in herbivorous insects include the effects of variability in host-plant foliage quality? Am Nat. 2005;165(1):16–31.

[27] Fuller E, Elderd BD, Dwyer G. Pathogen persistence in the environment and insect-baculovirus interactions: disease-density thresholds, epidemic burnout and insect outbreaks. Am Nat. 2012;179(3).

[28] Elderd BD. Developing models of disease transmission: insights from ecological studies of insects and their baculoviruses. PLoS Pathog. 2013;9(6):e1003372.

[29] Dwyer G, Dushoff J, Elkinton JS, Levin SA. Pathogen-driven outbreaks in forest defoliators revisited: Building models from experimental data. Am Nat. 2000;156(2):105–120.

[30] Bratsun D, Volfson D, Tsimring LS, Hasty J. Delay-induced stochastic oscillations in gene regulation. P Natl Acad Sci USA. 2005;102(41):14593–14598.

[31] Hunter AF. Traits that distinguish outbreaking and nonoutbreaking macrolepidoptera feeding on northern hardwood trees. Oikos. 1991;60:275–282.

[32] Páez D, Fleming-Davies A, Dwyer G. Effects of pathogen exposure on life-history variation in the gypsy moth (*Lymantria dispar*). J Evolution Biol. 2015;28(10):1828–1839.

[33] Páez DJ, Dukic V, Dushoff J, Fleming-Davies A, Dwyer G. Eco-evolutionary theory and insect outbreaks. Am Nat. 2017;189(6):616–629.

[34] Fleming-Davies AE, Dwyer G. Phenotypic Variation in Overwinter Environmental Transmission of a Baculovirus and the Cost of Virulence. Am Nat. 2015;186(6):797–806.

[35] Eakin L, Wang M, Dwyer G. The effects of the avoidance of infectious hosts on infection risk in an insect-pathogen interaction. Am Nat. 2014;185(1):100–112.

[36] Woods SA, Elkinton JS. Bimodal patterns of mortality from nuclear polyhedrosis-virus in gypsy-moth (*Lymantria-dispar*) populations. J Invertebr Pathol. 1987;50:151–157.

[37] Dwyer G, Elkinton JS. Host dispersal and the spatial spread of insect pathogens. Ecology. 1995;76(4):1262–1275.

[38] Johnson DM, Liebhold AM, Bjornstad ON, McManus ML. Circumpolar variation in periodicity and synchrony among gypsy moth populations. J Anim Ecol. 2005;74(5):882–892.

[39] Elkinton JS, Liebhold AM. Population dynamics of gypsy moth in North America. Annu Rev Entomol. 1990;35:571–596.

[40] Elderd BD, Rehill BJ, Haynes KJ, Dwyer G. Induced plant defenses, host–pathogen interactions, and forest insect outbreaks. P Natl Acad Sci USA. 2013;110(37):14978–14983.

[41] Poon LL, Song T, Rosenfeld R, Lin X, Rogers MB, Zhou B, et al. Quantifying influenza virus diversity and transmission in humans. Nat Genet. 2016;48(2):195–200.

[42] Zwart MP, Hemerik L, Cory JS, de Visser JAGM, Bianchi FJJA, Van Oers MM, et al. An experimental test of the independent action hypothesis in virus-insect pathosystems. Proc R Soc Lond B. 2009;276(1665):2233–2242.

[43] Abel S, zur Wiesch PA, Davis BM, Waldor MK. Analysis of bottlenecks in experimental models of infection. PLoS Pathog. 2015;11(6):e1004823.

[44] Lorenzo-Redondo R, Fryer HR, Bedford T, Kim EY, Archer J, Pond SLK, et al. Persistent HIV-1 replication maintains the tissue reservoir during therapy. Nature. 2016;530(7588):51+. doi:10.1038/nature16933.

[45] Varin C, Reid N, Firth D. An overview of composite likelihood methods. Statistica Sinica. 2011; p. 5–42.

[46] McVean G, Awadalla P, Fearnhead P. A coalescent-based method for detecting and estimating recombination from gene sequences. Genetics. 2002;160(3):1231–1241.

[47] Berger JO, Liseo B, Wolpert RL, et al. Integrated likelihood methods for eliminating nuisance parameters. Stat Sci. 1999;14(1):1–28.

[48] McDonald JH, Kreitman M. Adaptive protein evolution at the Adh locus in Drosophila. Nature. 1991;351(6328):652.

[49] Cingolani P, Platts A, Coon M, Nguyen T, Wang L, Land SJ, et al. A program for annotating and predicting the effects of single nucleotide polymorphisms, SnpEff: SNPs in the genome of *Drosophila melanogaster* strain w1118; iso-2; iso-3. Fly. 2012;6(2):80–92.

[50] R Core Team. R: A Language and Environment for Statistical Computing; 2012.

[51] Abdi H. In: Salkind N, editor. Bonferroni and Šidák corrections for multiple comparisons. Sage Thousand Oaks, CA; 2007. p. 103–107.
